# Online error rate control for platform trials

**DOI:** 10.1002/sim.9733

**Published:** 2023-04-02

**Authors:** David S. Robertson, James M. S. Wason, Franz König, Martin Posch, Thomas Jaki

**Affiliations:** a MRC Biostatistics Unit, School of Clinical Medicine, University of Cambridge, UK; b Population Health Sciences Institute, Faculty of Medical Sciences, Newcastle University, UK; c Section of Medical Statistics, Medical University of Vienna, Austria; d University of Regensburg, Germany

**Keywords:** multiple testing, online hypothesis testing, platform trial, type I error rate

## Abstract

Platform trials evaluate multiple experimental treatments under a single master protocol, where new treatment arms are added to the trial over time. Given the multiple treatment comparisons, there is the potential for inflation of the overall type I error rate, which is complicated by the fact that the hypotheses are tested at different times and are not necessarily pre-specified. Online error rate control methodology provides a possible solution to the problem of multiplicity for platform trials where a relatively large number of hypotheses are expected to be tested over time. In the online multiple hypothesis testing framework, hypotheses are tested one-by-one over time, where at each time-step an analyst decides whether to reject the current null hypothesis without knowledge of future tests but based solely on past decisions. Methodology has recently been developed for online control of the false discovery rate as well as the familywise error rate (FWER). In this paper, we describe how to apply online error rate control to the platform trial setting, present extensive simulation results, and give some recommendations for the use of this new methodology in practice. We show that the algorithms for online error rate control can have a substantially lower FWER than uncorrected testing, while still achieving noticeable gains in power when compared with the use of a Bonferroni correction. We also illustrate how online error rate control would have impacted a currently ongoing platform trial.

## 1 Introduction

There is a strong need to conduct high-quality evaluations of new interventions aimed at improving health of patients. The highest quality of evidence comes from Randomised Controlled Trials (RCTs). However, the cost of RCTs is high and increasing, leading to the very high costs of bringing new drugs to market [1] and evaluation of other types of intervention [2]. This has led to focus on methods that can improve the operational and statistical efficiency of conducting clinical trials.

One important class of efficient approaches are platform trials [3–5]. Platform trials set up an infrastructure that allows evaluation of multiple intervention arms under a single master protocol. Platform trials may use an adaptive design to drop non-promising intervention arms early and can add new interventions as they become available for evaluation. Some interventions can also be evaluated only within particular patient subgroups if warranted. These properties all can lead to increased efficiency [3, 6, 7]. However, they also introduce challenges, both operational [8] and statistical [9].

One statistical challenge, given the multiple treatment comparisons, is control of type I error rates. Although the need for formal control of type I error rates in trials of distinct treatments is controversial [10–16], it may be required in some regulatory settings and desirable in other situations where the treatments are related in some way. In a platform trial, controlling type I error rates is complicated by the fact that the hypotheses are tested at different times and are not all pre-specified.

Online error rate control methodology provides a possible solution to the problem of multiplicity for platform trials where multiple treatments are tested over time [17, 18]. In the online multiple hypothesis testing framework, hypotheses are tested one-by-one over time, where at each time-step a decision is made whether to reject the current null hypothesis without knowledge of future tests but based solely on past decisions. Methodology has recently been developed for online control of the false discovery rate (FDR) [19–24] as well as the familywise error rate (FWER) [25], so that the relevant error rate is controlled at all times throughout the trial.

In order to apply online error rate control methodology to platform trials and evaluate its performance, there are some general features specific to this setting that need to be explored. Firstly, one key consideration is the total number of hypothesis tests that is reasonable to envisage for a platform trial. Thus far, most of the literature on online testing has tended to focus on applications with a very large (≥ 1000) number of hypotheses. Secondly, many existing algorithms [19–22, 25] for online FDR or FWER control assume independence between the *p*-values (or equivalently, the test statistics). However, in the platform trial setting there is a positive dependence induced by shared control arm information, and so potential inflation of the relevant type I error rate needs to be considered. Lastly, in the clinical trial setting the usual error rate considered (at least for confirmatory trials) is the FWER [26, 27]. Hence, it would be useful to see how the FWER is impacted when using methods that control the FDR.

Our aim in this paper is to demonstrate the advantages and disadvantages of using online error rate control methodology in the platform trial setting through an extensive simulation study looking at varying numbers of treatment arms, patterns of expected treatment responses and arm entry times, as well as assumptions about the upper bound on the total number of experimental treatments to be tested.

In Section 2, we introduce the different algorithms for online error rate control that we will explore, and then in Section 3 we describe the idealised platform trial set-up that we use for the simulations and the different simulation scenarios. Section 4 provides the simulation results, focusing on testing treatments one-by-one (Section 4.1) as well as in batches (Section 4.2). In Section 5 we present a case study based on the STAMPEDE platform trial [28] of therapies for prostate cancer. We conclude with recommendations and discussion in Section 6.

## 2 Algorithms for online error rate control

**Figure 1:**
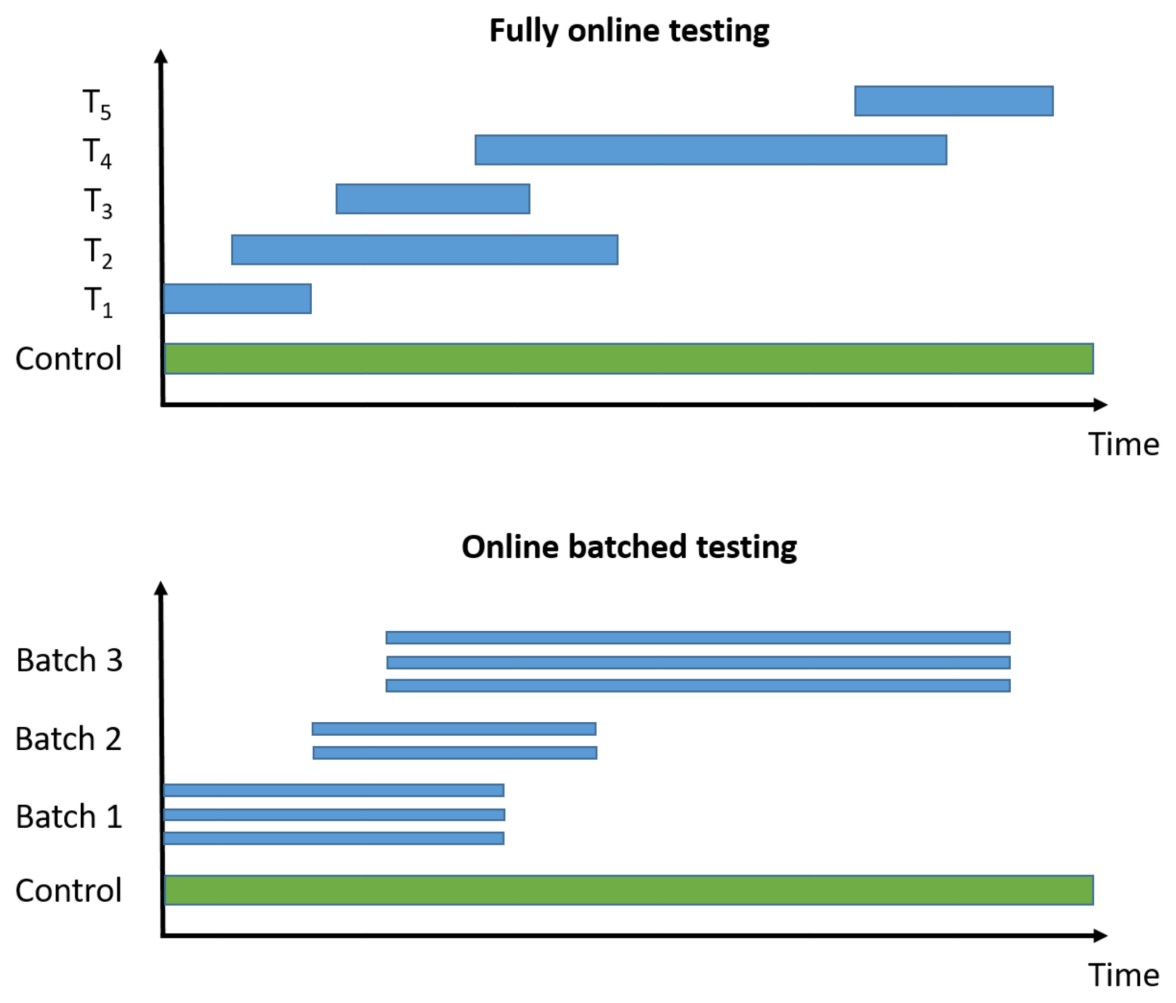
Illustration of fully online testing and online batched testing for a platform trial testing experimental treatments *T_i_* against a common control. For the online testing procedures, a hypothesis test can be conducted whenever new results become available (i.e. whichever treatment arm is the *i*-th one to be analysed is tested at level *α_i_*). For offline methods (e.g. the Benjamini-Hochberg procedure), testing can only occur when all the trial data are available.

We now briefly introduce the online multiple hypothesis testing algorithms that we consider, classified into those for fully online testing (i.e. where each hypothesis is tested one after another) and those for online batched testing (i.e. where groups of hypotheses are available to be tested at the same time). An illustration of the difference is given in Figure 1. All of the algorithms require a pre-specified significance level *α*, as well as a sequence of non-negative numbers *γ_i_* that sum to 1. We provide references giving further details of the algorithms for the interested reader. The algorithms described below are also available to use via the onlineFDR R package [29].

### 2.1 Fully online testing

The algorithms for fully online testing output adjusted testing levels *α_i_* for testing the null hypothesis *H_i_*, i.e. *H_i_* is rejected if the associated *p*-value *p_i_* satisfies *p_i_* ≤ *α_i_*.

- *LOND*: One of the first online testing algorithms proposed, LOND (Levels based on Number of Discoveries) provably controls the FDR for independent [19] and positively dependent *p*-values [23]. In LOND, the *α_i_* are calculated by simply multiplying the sequence *αγ_i_* by the number of discoveries (i.e. the number of rejected hypotheses) that have been made thus far.
- *LORD*: Another early online testing algorithm, LORD (Levels based on Recent Discovery) provably controls the FDR for independent *p*-values [19, 20]. LORD takes advantage of so-called “alpha-investing”, in which hypothesis tests costs some amount from the error budget (or “alpha-wealth”), but a discovery earns some of the error budget back. The *α_i_* in LORD depend not only on how many discoveries have been made but also on the timing of these discoveries. The more recent discoveries there are, the higher the alpha-wealth will be.
- *SAFFRON*: Proposed as an improvement to LORD, the SAFFRON (Serial estimate of the Alpha Fraction that is Futilely Rationed On true Null hypotheses) algorithm provably controls the FDR for independent *p*-values [21]. Intuitively, SAFFRON focuses on the stronger signals in an experiment (i.e. the smaller *p*-values). By never rejecting weaker signals (i.e. larger *p*-values), SAFFRON preserves alpha-wealth. When a substantial fraction of null-hypotheses are false, SAFFRON will often be more powerful than LORD.
- *ADDIS*: Proposed as an additional improvement to both LORD and SAFFRON, the ADDIS (ADaptive algorithm that DIScards conservative nulls) algorithm provably controls the FDR for independent *p*-values [25]. Intuitively, ADDIS builds on the SAFFRON algorithm by potentially investing alpha-wealth more effectively through the discarding of the weakest signals (i.e. the largest *p*-values) in a principled way. This procedure can gain appreciable power if the null *p*-values are conservative, i.e. stochastically larger than the uniform distribution.
- *ADDIS-spending*: Unlike all of the algorithms mentioned above, ADDIS-spending provably controls the FWER (in the strong sense) for independent *p*-values [22]. ADDIS-spending shares similarities with ADDIS in setting thresholds based on the size of the *p*-values for hypotheses to be selected for testing.

### 2.2 Online batched testing

The algorithms for online batched testing use well-known offline procedures for FDR control (i.e. procedures that require all of the *p*-values to be known before testing) for each batch, in such a way that the FDR is controlled across all of the batches over time. Three related algorithms were proposed by Zrnic et al. [24]:

- *BatchBH*: provably controls the FDR when the *p*-values are independent within and across batches. BatchBH runs the Benjamini-Hochberg (BH) procedure on each batch, where the values of *α_i_* depend on the number of previous discoveries.
- *BatchPRDS*: a modification of BatchBH, which provably controls the FDR when the p-values in one batch are positively dependent, and independent across batches.
- *BatchStBH*: provably controls the FDR when the *p*-values are independent within and across batches. BatchStBH runs the Storey Benjamini-Hochberg (StBH) procedure on each batch, where the values of *α_i_* depend on the number of previous discoveries. The StBH procedure can potentially make more rejections than BH by adapting to the (estimated) number of non-nulls.

## 3 Simulation study

We now describe the platform trial set-up (Section 3.1) used in our simulation study, as well as the simulation scenarios (Section 3.2) that we explore.

### 3.1 Platform trial set-up

Consider an idealised platform trial that eventually tests a total of *K* experimental treatments *T*_1_, …, *T_K_* against a common control *T*_0_ (see Figure 13 for a graphical example). Let *μ_i_* denote the expected response for treatment *T_i_*, and define the treatment effect *θ_i_* = *μ_i_* − *μ*_0_ for *i* ∈ {1, …, *K*}. The null hypotheses of interest are given by *H*_0*i*_ : *θ_i_* ≤ 0 with corresponding alternatives *H*_1*i*_ : *θ_i_* > 0, which we test at error level *α* (for either FWER or FDR). Let *n_i_* denote the pre-specified sample size for each experimental treatment, and for simplicity assume that an equal number of patients are eventually allocated to each experimental arm (i.e. *n*_1_ = … = *n_K_* = *n*). We use the simplifying assumption that an experimental treatment continues in the trial until *n* patients have been allocated to that arm and their outcomes observed. The experimental treatment is then formally tested for effectiveness by comparing with *concurrent* controls (i.e. only the outcomes from patients allocated to the control group during the time that the experimental treatment was active in the trial). We return to the issue of conducting interim analyses in Section 6.

We assume that the observation *X_ij_* from patient *j* on treatment *T_i_* is distributed (at least asymptotically) as *X_ij_* ∼ *N* (*μ_i_*, *σ*^2^), where *σ* is known. The mean response for patients on experimental treatment *T_i_* (*i* > 0) is then given by ${\bar{X}}_{i}=\frac{1}{n}\sum_{j=1}^{n}X_{ij}$, with corresponding concurrent control mean ${\bar{X}}_{0}^{\left(i\right)}=\frac{1}{N_{0}\left(i\right)}\sum_{j\in T_{i}}X_{0j}$, where *N*_0_(*i*) and $T_{i}$ denote the number and index set, respectively, of the control observations that are concurrent with *T_i_*. The comparisons between the experimental treatments and the control are based on normally-distributed test-statistics

$$Z_{i}=\frac{{\bar{X}}_{i}-{\bar{X}}_{0}^{\left(i\right)}}{\sigma \sqrt{\frac{1}{N_{0}\left(i\right)}+\frac{1}{n}}}∼N\left(\frac{\theta_{i}}{\sigma \sqrt{\frac{1}{N_{0}\left(i\right)}+\frac{1}{n}}},1\right)$$

where ${\bar{X}}_{i}-{\bar{X}}_{0}$ is the observed difference in means. The corresponding one-sided *p*-value for treatment *T_i_* is given by *p_i_* = 1 − Φ(*Z*), where Φ is the standard normal cdf.

We assume for simplicity that experimental treatment *T_i_* enters the trial at time *t_i_*, and remains in the trial until time (*t_i_* + *r*). The platform trial starts at time 0 and finishes once all *K* experimental treatments have been tested. Hence the temporal structure of the platform trial can be described by the value of *r* and the vector ***t*** = (*t*_1_, …, *t_K_*). Without loss of generality, we impose the restriction that *t*_1_ ≤ … ≤ *t_K_*. We assume that *n*/*r* patients are allocated to each arm (including the control) per unit time, where *r* is chosen so that *n*/*r* is an integer.

### 3.2 Simulation scenarios

In our simulation study, we consider *K* ∈ {5, 10, 15, 20}, and set *α* = 0.025, *n* = 50, *μ*_0_ = 0, *σ* = 1 and *r* = 10. Table 1 gives an overview of the scenarios, algorithms and performance metrics used in the simulation study, with further details given below.

We consider the following scenarios for the treatment means *μ_i_*, *i* ∈ {1, …, *K*}:

1. Global null: *μ*_1_ = … = *μ_K_* = 0
2. Fixed means: *m* effective treatments which are either all tested at the start or the end of the platform trial, or are tested in a random order. We set *μ_i_* = 0.5 for *i* ∈ *I* and *μ_i_* = 0 otherwise, where
  *I* = {*i* : 1 ≤ *i* ≤ *m*} (Early)
  *I* = {*i* : *K* − *m* + 1 ≤ *i* ≤ *K*} (Late)
  *I* is a set of *m* values drawn randomly from {1, …, *K*} without replacement (Random)
  for *m* ∈ {1, *K*/5 + 1, 2*K*/5 + 1}.
3. Staircase scenario:
  *μ_i_* = (*i* − [*K*/2])/*K* (Increasing)
  *μ_i_* = ([*K*/2] − *i* + 1)/*K* (Decreasing)
  *μ_i_* is drawn randomly from the set {(*i*−[*K*/2])/*K* : 1 ≤ *i* ≤ *K*} without replacement (Random)

We also consider the following patterns of arm entry times ***t***:

1. All arms at once (offline testing): *t_i_* = 0
2. Batches of size *b*: *t_i_* = *r*(*j* − 1) for *i* ∈ {*b*(*j* − 1) + 1, …, *bj*} and *j* ∈ {1, …, *K*/*b*}, where *b* = 5
3. Staggered starts: *t_i_* = *r*(*i* − 1)/*s* for *s* ∈ {2, 5}
4. One-by-one (fully online): *t_i_* = *r*(*i* − 1)

Finally, we explore different assumptions about the upper bound, *N*_bound_, on the total number of experimental treatments to be tested. We consider *N*_bound_ ∈ {*K,* 2*K,* 5*K*}, i.e. considering the impact of more conservative choices of *N*_bound_. Note that *N*_bound_ should be chosen before the start of the platform trial. We return to the issue of what happens if *N*_bound_ *< K* in Section 6.

For each of the simulation scenarios described above, we compare the following algorithms for FWER and FDR control:

**FWER**: Bonferroni and ADDIS-spending.
**FDR**: ADDIS, SAFFRON and LOND. For batched entry times, we consider the BatchBH, BatchPRDS and BatchStBH procedures. We also use the standard Benjamini-Hochberg (BH) procedure as a comparator.

We give more details about the exact implementation of these algorithms in Section A of the Supporting web materials. Note that Bonferroni tests each hypothesis at level *α*/*N*_bound_ (as opposed to *α*/*K*), and the BH procedure is an offline procedure that requires all hypotheses to be tested at once.

For each simulation scenario, we report the following performance measures:

**Type I error rates**: FWER and FDR
**Power metrics**: Disjunctive power (probability of rejecting at least one non-null hypothesis) and Sensitivity (proportion of non-null hypotheses that are rejected)

The use of disjunctive power in our comparisons reflects how it is the power analog of FWER. However, as pointed out by a reviewer, arguably the most relevant definition of ‘power’ when making multiple comparisons is the power to detect a given treatment effect and hence sensitivity may be a more relevant metric to use. We note that for simplicity we have kept type I error and power metrics separate in the results that follow. Alternatively, it could be useful to consider a metric that simultaneously incorporates both power and type I error rates. However, to combine both metrics in this way would require the specification of the relative costs of making type I and type II errors, which would vary across different stakeholders.

## 4 Simulation results

To start with, we consider the type I error rate of the different algorithms, in particular checking whether the FDR is controlled. This is of interest because apart from LOND, all of the online testing algorithms only provably control the FDR under independence of the *p*-values. Figure B1 in Section B.1 of the Supporting web materials shows the FDR under the global null (and hence the FWER = FDR), for varying patterns of arm entry times and *N*_bound_ = *K*. Note that the global null maximises the FWER for multi-arm multi-stage (MAMS) designs [30]. Using uncorrected testing leads to a highly inflated FDR/FWER above the nominal 2.5%, which can be as high as 40% when *K* = 20. In contrast, the online testing algorithms have a FDR/FWER which is equal to or below the nominal level. The one exception is SAFFRON, which has a slight inflation of the FDR/FWER to around 3%.

### 4.1 Fully online setting

Continuing with the FDR and now focusing on the fully online setting, Figure B2 in Section B.2 of the Supporting web materials shows the worst and best case FDR (corresponding to all the effective treatments appearing Early and Late, respectively) for fixed means and different numbers of effective treatments, with *N*_bound_ = *K*. Again we see how uncorrected testing can lead to a highly inflated FDR, although this inflation reduces as the number of effective treatments increases. This time, all the online testing procedures control the FDR at or below the nominal 2.5%, but they can be noticeably conservative. This is the case for LORD, as well as ADDIS-spending for (*K*/5 + 1) and (2*K*/5 + 1) effective treatments. Note though that the Bonferroni correction is also highly conservative in these settings.

**Figure 2:**
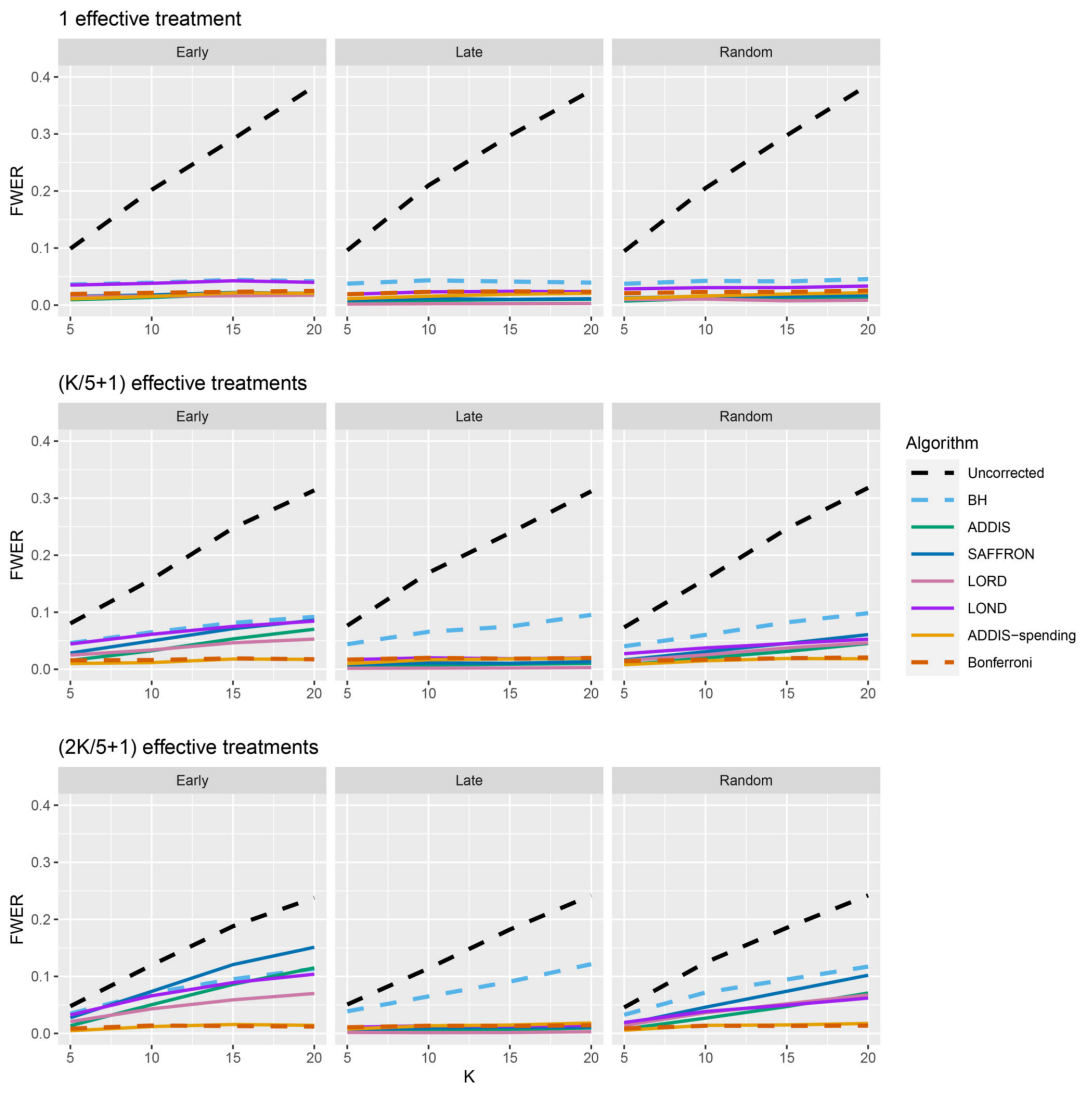
FWER for fixed means and different numbers of effective treatments, with *N*_bound_ = *K*.

We now move on to examining the FWER for the different procedures. Of course, the FDR-controlling algorithms would not be expected to also control the FWER in general, but it is of interest to see how large any inflation in the FWER can be. In general, the magnitude of the inflation depends critically on the effect sizes chosen for the alternative hypotheses. Figure 2 shows the FWER under the early and late scenarios, as well as the FWER under the random ordering of the effective treatments, all with *N*_bound_ = *K*. When there is only 1 effective treatment the FWER is controlled by all the online testing algorithms, but (apart from LOND) they are even more conservative than Bonferroni. A possible explanation for this observation is that the test statistics are independent in the fully online setting, and the Bonferroni correction is not as highly conservative under independence. Under the global null (for example), the probability of at least one error when using Bonferroni is 1 − (1 − *α*/*K*)^*K*^ and this is close to *α* for small *α*.

When there are (*K*/5 + 1) effective treatments, the FWER is inflated above 2.5% for all the online testing procedures (apart from ADDIS-spending) when *K* > 5 in the Early scenario and *K* > 15 in the Random scenario. The LOND algorithm has the largest inflation in the Early scenario, which is similar to the inflation seen using the BH procedure. Finally, when there are (2*K*/5+1) effective treatments, there is FWER inflation for all the online algorithms (except for ADDIS-spending) when *K* > 5 in the Early scenario and *K >* 10 in the Random scenario. This time, SAFFRON has the highest inflation, which can be higher than BH in the Early scenario, although still noticeably lower than uncorrected testing. Note that in the random setting, for both (*K*/5 + 1) and (2*K*/5 + 1) effective treatments, when *K* = 15 the FWER of the online testing procedures (apart from SAFFRON) is controlled at 5%.

**Figure 3:**
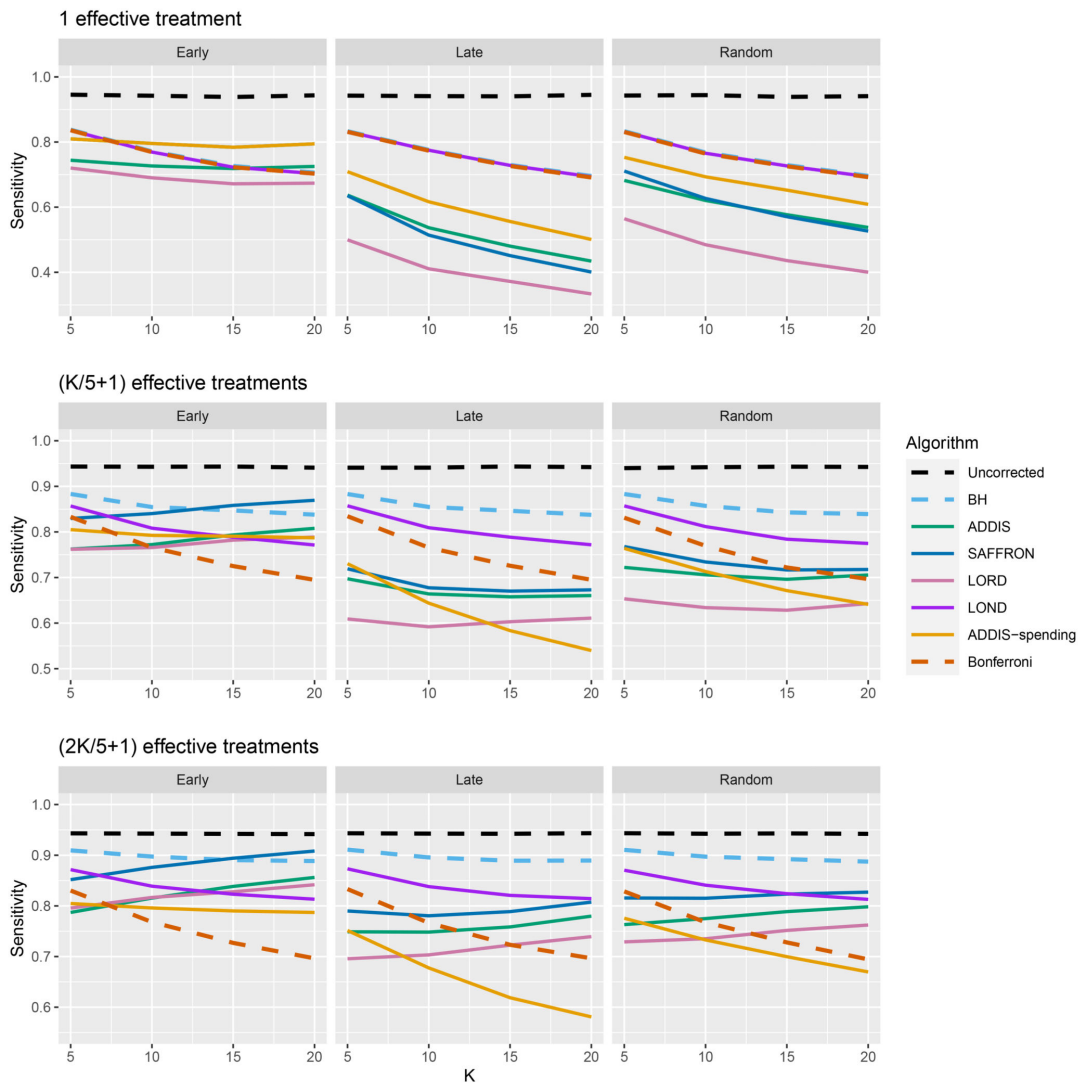
Sensitivity for fixed means and different numbers of effective treatments, with *N*_bound_ = *K*. Note that the *y*-axis scales differ as the number of effective treatments vary for readability.

The other side of the story is the power of the trial, and to start with we focus on sensitivity as a measure of the proportion of the truly effective treatments that are declared efficacious. Figure 3 shows the sensitivities for the Early, Late and Random scenarios, again with *N*_bound_ = *K*. For ease of reference, we also include plots showing the Sensitivity and FWER together in Figures B3–B5 in Section B.2 of the supporting web materials. With only 1 effective treatment, the sensitivity of all of the online algorithms apart from LOND is substantially lower than using Bonferroni. Even in the best case (Early scenario), only SAFFRON and ADDIS-spending have a higher sensitivity than Bonferroni when *K* ≥ 10. When there are (*K*/5 + 1) effective treatments, again the sensitivities of all of the online algorithms apart from LOND are lower than Bonferroni in the Late and Random scenarios. LOND maintains a sensitivity roughly halfway that between the Bonferroni and BH procedures. In the best case (Early scenario), SAFFRON has a higher sensitivity than Bonferroni for *K* > 5 (and even BH for *K* ≥ 15), while the other online algorithms also achieve a higher sensitivity for *K* > 10. Finally, with (2*K*/5+1) effective treatments, we again see that LOND maintains a sensitivity roughly halfway that between the Bonferroni and BH procedures. Under the Random scenario, SAFFRON, ADDIS and LORD have a higher sensitivity than Bonferroni for *K* > 5, *K* > 10 and *K* ≥ 15, respectively. In the worst case (Late scenario), the equivalent values of *K* are *K* ≥ 10, *K* > 10 and *K* > 15, respectively. Finally, in the best case (Early scenario), all the online testing algorithms outperform Bonferroni for *K* ≥ 10.

**Figure 4:**
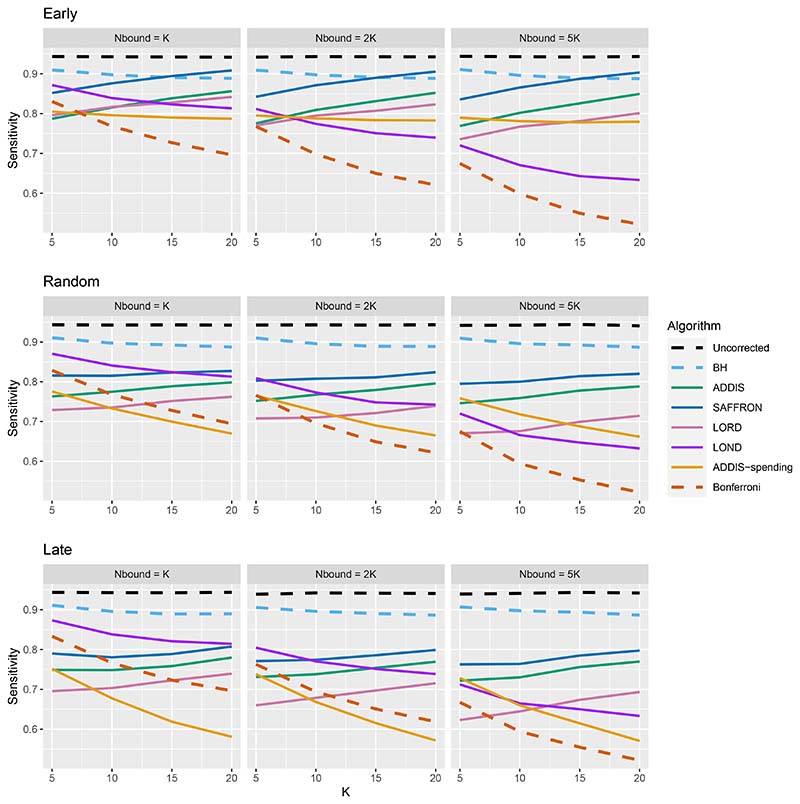
Sensitivity for fixed means and (2*K*/5 + 1) effective treatments, with varying *N*_bound_.

Thus far we have used an upper bound *N*_bound_ = *K*, i.e. exactly equal to the number of treatments that are tested. In practice, this is unlikely to be known prior to the platform trial starting, and a more conservative (i.e. higher) value of *N*_bound_ would be used. Figure 4 shows the effect of increasing the value of *N*_bound_ on the sensitivity of the algorithms, where there are (2*K*/5 + 1) effective treatments. The key takeaway here is that as *N*_bound_ increases, the sensitivity of Bonferroni, LOND and LORD noticeably decreases, with Bonferroni having the largest relative decrease, whereas the sensitivity of all the other algorithms remains virtually unchanged. This means that there is a greater advantage in using online algorithms (in terms of sensitivity) compared with Bonferroni as *N*_bound_ increases. For example, in the extreme case where *N*_bound_ = 5*K*, then under a random ordering of treatment means, all of the online algorithms outperform Bonferroni, with the relative advantage increasing with *K*. The same is seen for the more realistic scenario of *N*_bound_ = 2*K*, apart from LORD for *K <* 10.

**Figure 5:**
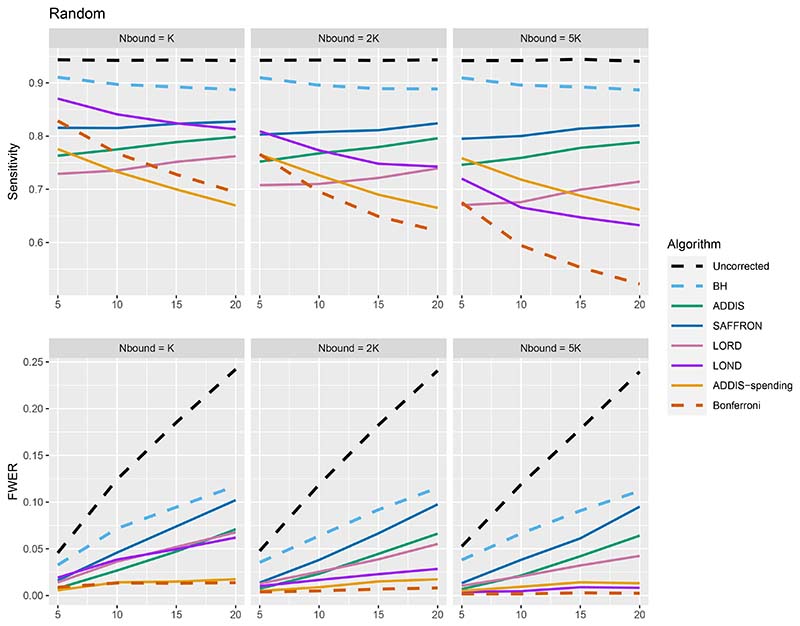
Sensitivity with corresponding FWER for random fixed means and (2*K*/5+1) effective treatments, with varying *N*_bound_.

In terms of the effect of *N*_bound_ on the FWER, Figure 5 shows the trade-off between sensitivity and the FWER under a random ordering of treatment means. (The results for the Early and Late scenarios are given in Figures B6 and B7 in Section B.2 of the Supporting web materials). We see that as *N*_bound_ increases, the FWER of Bonferroni, LOND and LORD decreases, whereas the FWER of the other algorithms remain virtually unchanged. Hence, when *N*_bound_ = 2*K*, LOND controls the FWER at 2.5% while still having substantial gains in power over Bonferroni. Meanwhile, the power gains of SAFFRON (and to a lesser extent, ADDIS) come at the cost of a large inflation of the FWER. However, we note that in order to be conservative it may still be useful to consider the FWER when *N*_bound_ = *K* since it represents a sort of ‘worst-case’ scenario where the assumed upper bound on the total number of treatments is actually reached.

**Figure 6:**
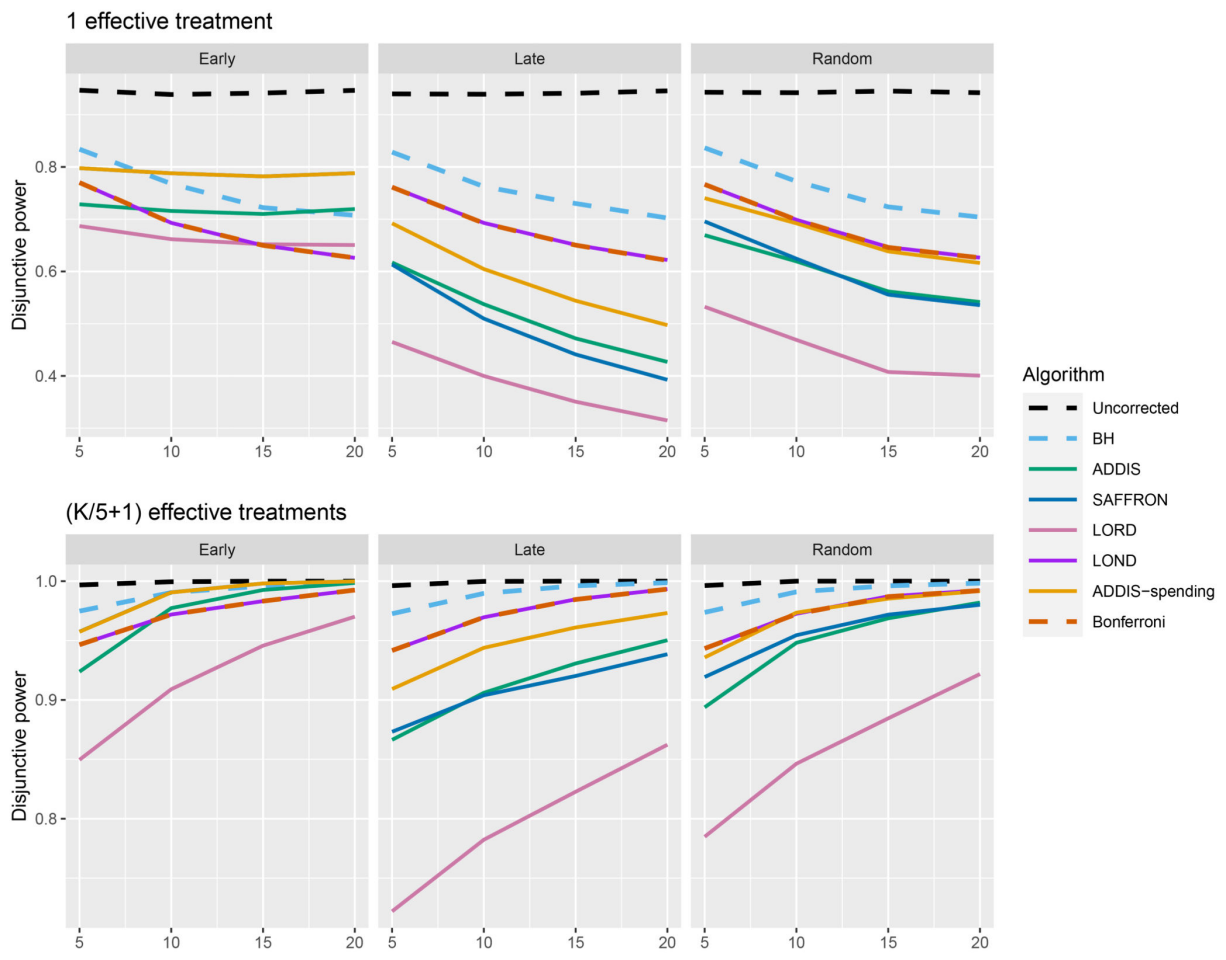
Disjunctive power for fixed means and different numbers of effective treatments, with *N*_bound_ = 2*K*.

Figure 6 shows the disjunctive power for 1 and (*K*/5+1) effective treatments, with *N*_bound_ = 2*K* (results for (2*K*/5 + 1) effective treatments are not shown since most methods have a disjunctive power very close to 1). Using this power metric, the online algorithms all have substantially lower power than Bonferroni in the Random and Late scenarios, apart from LOND (and ADDIS-spending for randomly ordered means). Only in the Early scenario do we see advantages for ADDIS-spending and (to a lesser extend) ADDIS, particularly when there is only 1 effective treatment.

**Figure 7:**
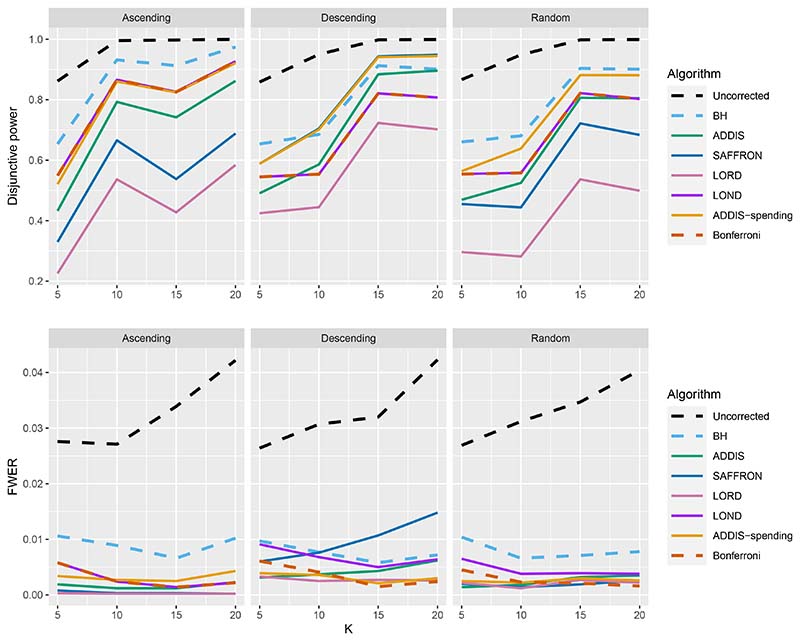
Disjunctive power and FWER for the staircase scenarios. Here *N*_bound_ = 2*K* for the disjunctive power plots and *N*_bound_ = *K* for the FWER plots.

Finally, we consider the impact of conservative nulls (i.e. the staircase scenario) in the fully online setting. A-priori we would expect ADDIS and ADDIS-spending to perform better here. Due to the treatment means used, the sensitivity of all algorithms is low (< 50%) and hence we only focus on the disjunctive power (the results for sensitivity are available in Section B.2 of the Supporting web materials, Figure B8). Figure 7 shows the results for the staircase scenario, where *N*_bound_ = 2*K* for the disjunctive power plots and *N*_bound_ = *K* for the FWER plots. In this setting and for this power metric, ADDIS-spending performs particularly well with comparable disjunctive power to Bonferroni in the worst case (Ascending) and noticeable increases in power under the Random and Descending staircase scenarios. Note that the FWER of all the procedures (except Uncorrected testing) control the FWER below the nominal 2.5% level.

In summary, we have seen that the performance of the fully online testing algorithms strongly depends on the number of effective treatments, the order in which they are tested in the trial, and the assumed upper bound *N*_bound_ on the total number of treatment arms. As would be expected, the power of the online algorithms increases when there is a relatively large number of effective treatments which appear early on in the trial. However, as is the case for offline FDR controlling procedures, the online FDR algorithms do not control the FWER, although the FWER is still noticeably lower than with uncorrected testing.

Overall, LOND is a robust choice across the scenarios considered, since it provably always make at least as many rejections as Bonferroni, which can sometimes translate into substantial sensitivity gains, while maintaining a reasonable FWER for all but the most extreme cases. For example, under (arguably) the most plausible setting of a random ordering of the truly effective treatment, when *N*_bound_ = 2*K* even when *N*_bound_ = 2*K* the FWER on LOND is below the nominal 2.5%. Even when *N*_bound_ = *K*, the FWER is still below 5% for *K* ≤ 15. In the staircase scenario though, ADDIS-spending performs surprisingly well in terms of disjunctive power, with a noticeably higher power than LOND and Bonferroni in the Random and Descending scenarios (while still maintaining FWER control). We return to our general recommendations in the Discussion (Section 6).

### 4.2 Online batched algorithms

**Figure 8:**
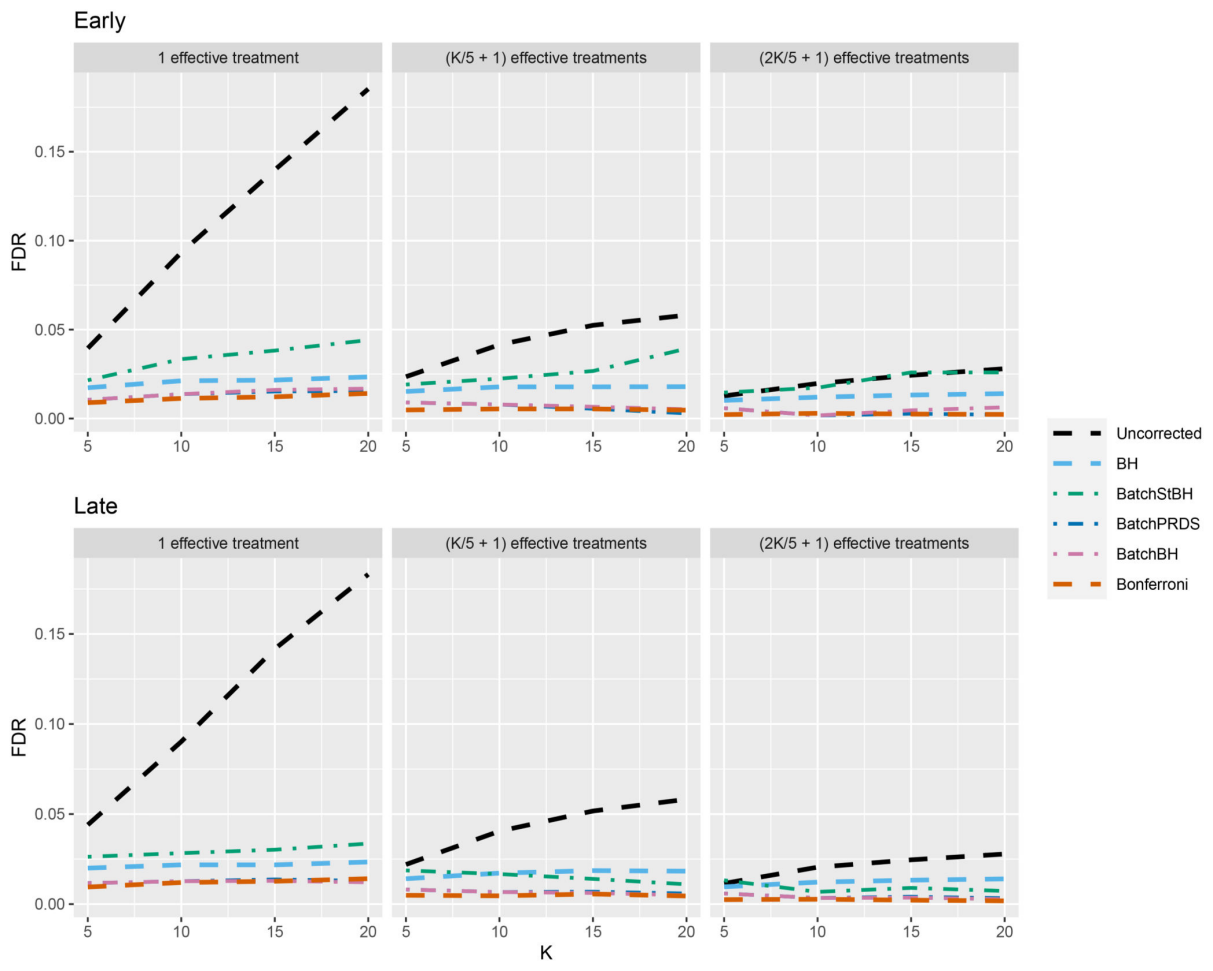
FDR for the online batched algorithms, with *N*_bound_ = *K*.

We now turn our attention to the batched setting (with a batch size of 5) in order to assess how the online batched algorithms (BatchBH, BatchPRDS and BatchStBH) compare with the fully online algorithms. Starting with the FDR, Figure 8 shows the worst and best case FDR (corresponding to the Early and Late scenarios, respectively) of the online batched algorithms when *N*_bound_ = *K*. We see that while BatchBH and BatchPRDS control the FDR below the nominal 2.5% in all the scenarios, BatchStBH has an inflated FDR when there is 1 effective treatment which approaches 5% in the worst case when *K* = 20. This inflation of the FDR of BatchStBH is theoretically justified, since BatchStBH only controls the FDR under independence of the *p*-values both within and across batches.

**Figure 9:**
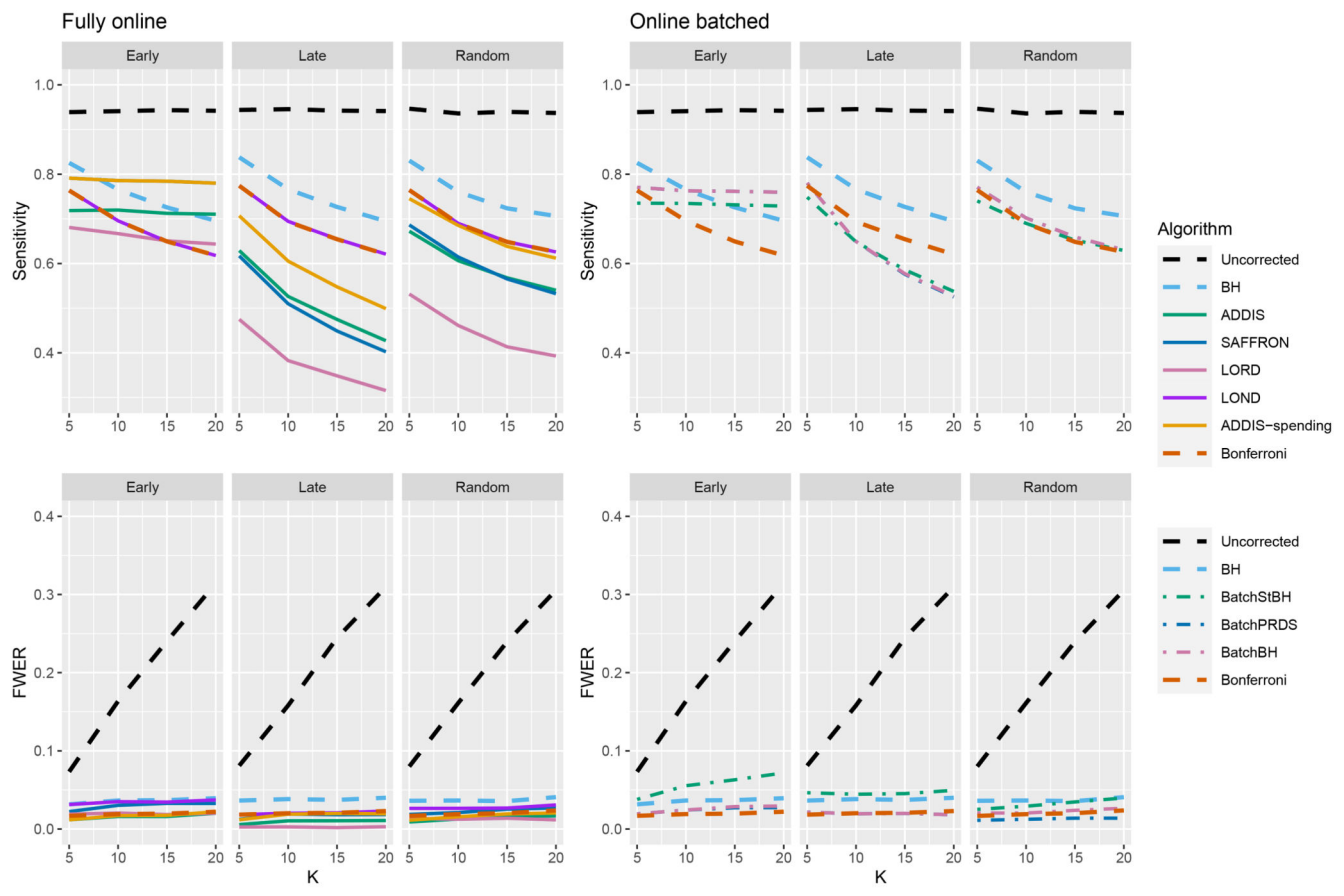
Comparison of sensitivity and FWER for fully online and online batched algorithms, with 1 effective treatment. Here *N*_bound_ = 2*K* for the sensitivity and *N*_bound_ = *K* for the FWER.

**Figure 10:**
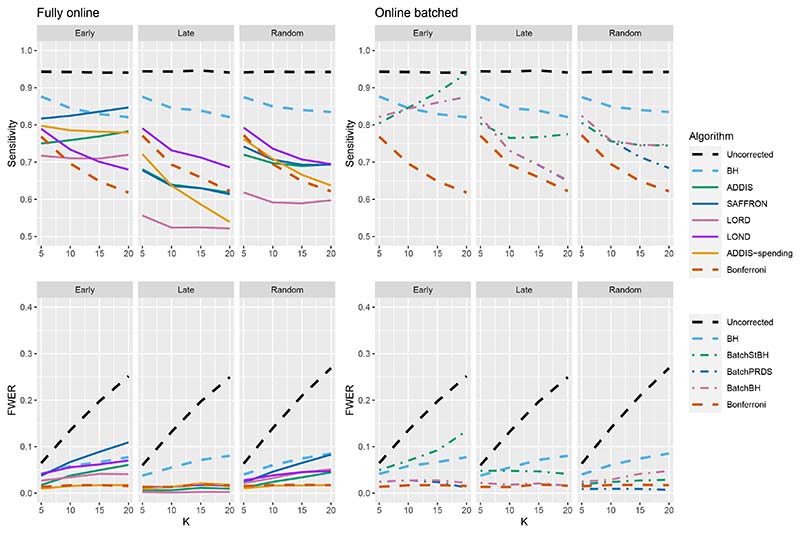
Comparison of sensitivity for fully online and online batched algorithms, with (*K*/5+1) effective treatments. Here *N*_bound_ = 2*K* for the sensitivity and *N*_bound_ = *K* for the FWER.

**Figure 11:**
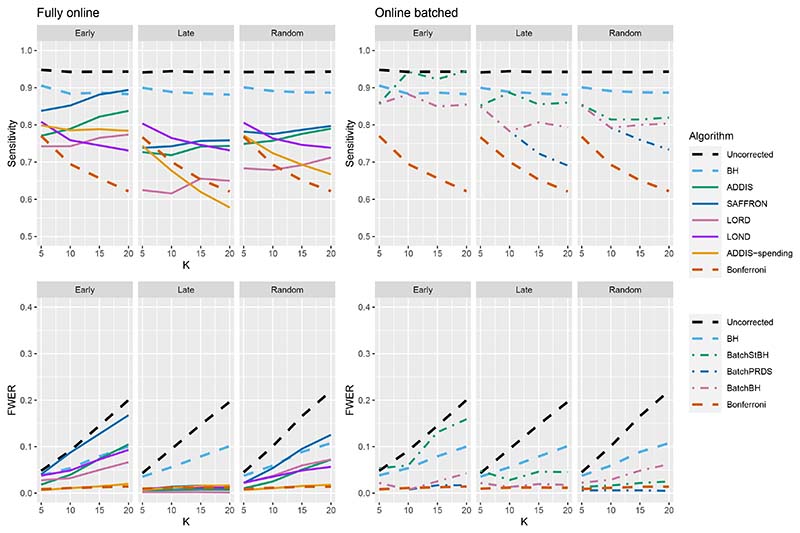
Comparison of sensitivity for fully online and online batched algorithms, with (2*K*/5 + 1) effective treatments. Here *N*_bound_ = 2*K* for the sensitivity and *N*_bound_ = *K* for the FWER.

Figures 9–11 compares the sensitivity and FWER for fully online and online batched algorithms, with *N*_bound_ = 2*K* for the sensitivity and *N*_bound_ = *K* for the FWER. Starting first with 1 effective treatment (Figure 9), there is no advantage in terms of sensitivity when using the online batched algorithms compared to LOND under the Late and Random scenarios, or ADDIS-spending and SAFFRON in the early scenario. In all scenarios, BatchStBH has a noticeably inflated FWER, particularly under the Early scenario. In contrast, BatchBH and BatchPRDS are reasonably close to the nominal 2.5% level.

When there are (*K*/5 + 1) effective treatments (Figure 10), there is a slight sensitivity advantage in using BatchBH and BatchStBH over LOND in the Random scenario for *K* > 10, with similar or lower FWER. In the Early scenario, BatchBH and BatchPRDS have similar sensitivity to SAFFRON but substantially lower FWER (of approximately 2.5%). Meanwhile, BatchStBH has a higher sensitivity than SAFFRON for *K* > 10, but at the cost of an even more inflated FWER. Finally, in the Late scenario, BatchBH and BatchPRDS have a slightly lower sensitivity than SAFFRON, whereas BatchStBH has a substantially higher sensitivity but at the cost of a noticeable inflation of the FWER.

When there are (2*K*/5 + 1) effective treatments (Figure 11), BatchPRDS has similar sensitivity to LOND but with a substantially lower FWER that is below the 2.5% level. BatchBH and BatchStBH have similar sensitivity to SAFFRON and ADDIS respectively when *K* ≥ 10, but BatchStBH has substantially lower FWER (approximately 2.5%) while BatchBH has similar FWER to ADDIS. In the Early scenario, BatchBH and BatchPRDS have a roughly similar sensitivity to SAFFRON, but with a substantially lower FWER (which as at or below 2.5% for BatchPRDS). BatchStBH has a higher sensitivity than SAFFRON (in fact approaching the level of uncorrected testing) but with a similarly highly inflated FWER. Lastly, in the Late scenario BatchPRDS has a lower sensitivity than LOND for *K* ≥ 10 although still noticeably higher than Bonferroni. BatchBH has a slightly higher sensitivity than LOND with a similar FWER. Meanwhile BatchStBH has a substantially higher sensitivity than LOND (or any of the other fully online algorithms) but again at the cost of a noticeably higher FWER.

In terms of disjunctive power, Figure B9 in Section B.3 of the Supporting web materials shows that all of the online batched algorithms have fairly similar power to ADDIS-spending across the different scenarios. However, as already seen this comes at the cost of a (potentially highly) inflated FWER for BatchStBH and to a lesser extent BatchBH.

**Figure 12:**
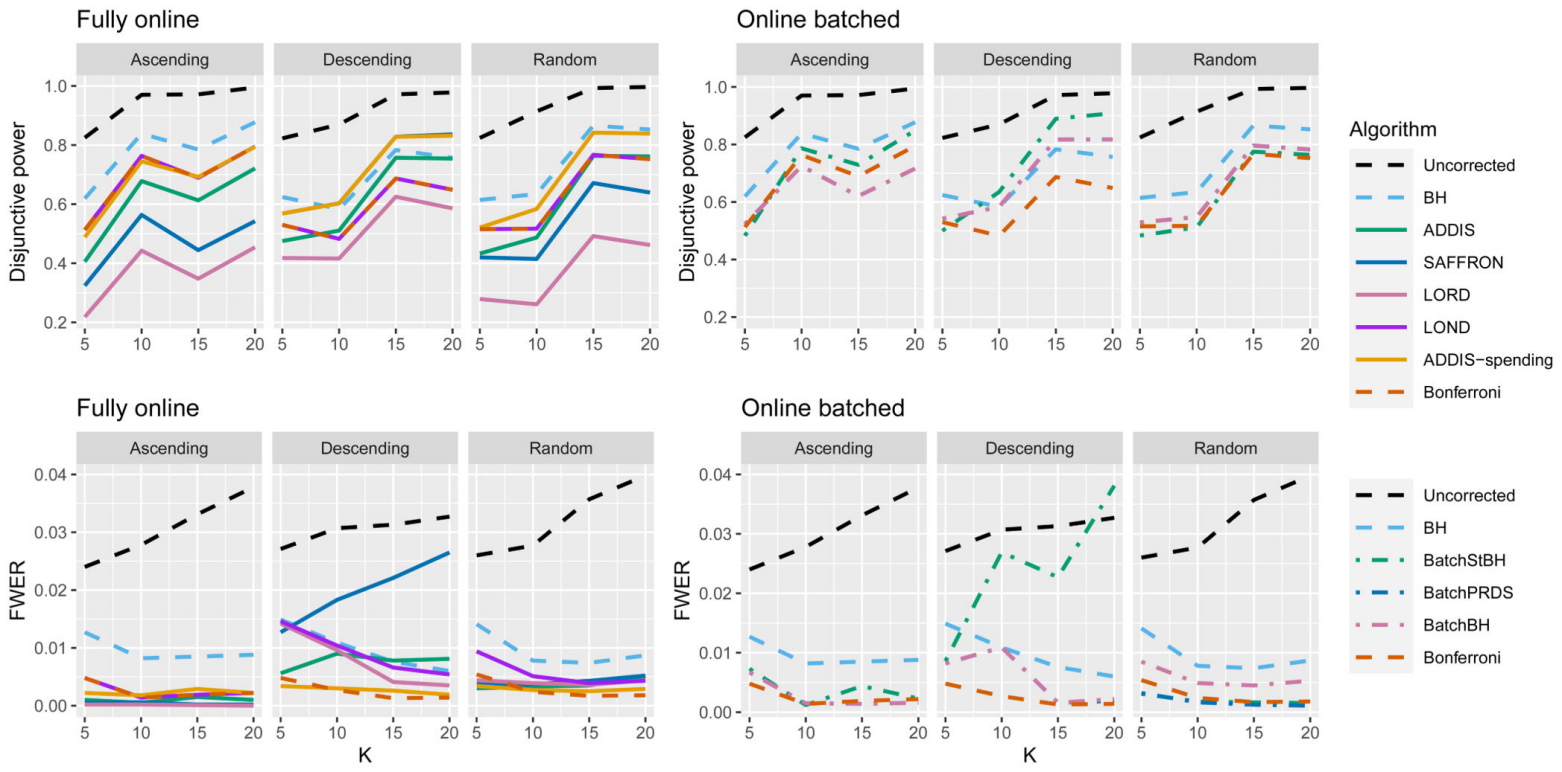
Comparison of the FWER (*N*_bound_ = *K*) and disjunctive power (*N*_bound_ = 2*K*) for fully online and online batched algorithms under the staircase scenario.

Finally, Figure 12 gives the results for the staircase scenario with conservative nulls, in terms of disjunctive power and FWER. We again see that the online batched algorithms tend to have similar disjunctive power to ADDIS-spending, although BatchStBH has slightly higher values in the Ascending and Descending scenarios and all the online batched algorithms have slightly lower values in the Random scenarios. As for the FWER, all the online batched algorithms control the FWER below the nominal 2.5%, except for BatchStBH under the Descending scenario.

Looking at the results for online batched testing as a whole, we see that the BatchPRDS algorithm is competitive when compared to LOND (and hence also Bonferroni) by having similar or higher power, in terms of sensitivity and disjunctive power, while still controlling the FWER at 2.5% across all the scenarios considered.

Finally, an alternative strategy to try to control the FDR (or FWER) in the batch setting is to apply the BH or Holm procedure to each batch. However, a repeated application of the BH (Holm) procedure each at level *α* will not in general control the FDR (FWER) at level *α* across batches. For example, in the extreme case of all batches being of size 1, then such a strategy using the Holm procedure would be identical to using uncorrected testing at level *α*. Nonetheless, we have evaluated the use of a repeated BH and a repeated Holm procedure for the batch setting, with the results found in Section B.3 of the Supporting information. There can be a highly inflated FDR and FWER for this strategy but also a substantial increase in sensitivity in some scenarios.

## 5 Case study: STAMPEDE trial

**Figure 13:**
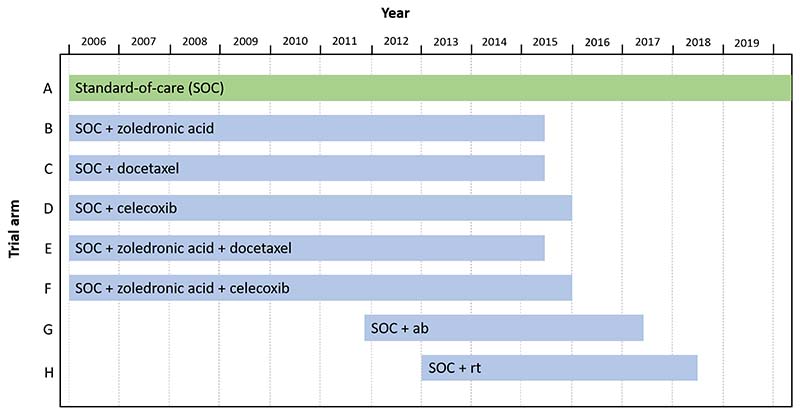
Schematic of the STAMPEDE trial. ab = abiraterone, rt = radiotherapy.

The STAMPEDE (Systemic Therapy for Advancing or Metastatic Prostate Cancer) trial is a flagship platform trial for research into the effect of systemic therapies for prostate cancer [28] on overall survival. The trial opened to accrual in October 2005 with 6 arms (A–F), including the control arm A, which was standard-of-care (SOC) hormone therapy. Figure 13 shows a schematic of the treatment comparisons that have already been reported from STAMPEDE. Two additional arms (G and H) were added to the trial in 2011 and 2013, respectively. Three arms (B, C and E) reported main analyses in 2015 [31], with two additional arms (D and F) reporting in 2015/16 [32]. Arm G reported main analyses in 2017 [33] and Arm H reported results in 2018 [34].

Table 2 shows the reported *p*-values (unadjusted for multiplicity) when comparing arms B to H with the SOC (arm A), as given in [31–34]. The dashed lines denote the 4 batches in the trial.

We now apply the offline and online testing algorithms to the observed *p*-values given above in Table 2, keeping the alphabetical ordering of *p*-values within the three batches. As a sensitivity analysis, we show what happens if the ordering of the *p*-values in the first batch changes in Table C2 in Section C of the Supporting web materials. We set the upper bound on the number of treatments *N*_bound_ = 20, i.e. twice as many arms that have already entered the STAMPEDE trial as of the end of 2021.

Table 3 shows which of the hypotheses corresponding to each trial arm can be rejected at level *α* ∈ {0.025, 0.05, 0.1}, as well as the current significance level *α*_8_ that would be used to test the next treatment arm after the 7 already evaluated in the trial. We consider larger values of *α* since this has been a suggestion for multi-arm trials [35] as a compromise between not correcting for multiplicity at all (as would be the case when running a series completely independent two-arm trials) and strict FWER control at 2.5%.

When *α* = 0.025, uncorrected testing rejects hypotheses C, E and G, but only BH and BatchStBH reject more than one hypothesis (C and G). All the other online algorithms as well as Bonferroni only reject hypothesis G, except for ADDIS and LORD which reject no hypotheses. When *α* = 0.05 the rejections made by the various algorithms remain the same, except now SAFFRON, BatchBH and BatchPRDS reject hypotheses C and G, while BatchStBH rejects hypotheses C, E and G. Finally, when *α* = 0.1 we see that BH, SAFFRON, BatchBH, BatchPRDS and BatchStBH all make the same rejections as Uncorrected testing. In terms of the current adjusted testing level *α*_8_, we see that ADDIS-spending, ADDIS and LORD have a smaller value than even Bonferroni. SAFFRON, LOND, BatchBH and BatchPRDS have a larger value of *α*_8_ than Bonferroni, but only BatchStBH has a higher value than Uncorrected testing.

## 6 Discussion

In this paper, we have shown how online multiple hypothesis testing can be applied to the platform trial setting to achieve overall control of type I errors. In many of the simulation scenarios, there were noticeable gains in sensitivity compared with using Bonferroni, although this has to be considered carefully with respect to the potential inflation of the FWER and how acceptable that may be to stakeholders (particularly from a regulatory viewpoint). However, in all cases the FWER was lower than Uncorrected testing, and so from that perspective, any of the online algorithms would offer an improvement.

We have focused on trial settings that ultimately test *N* ≥ 20 hypotheses, which is a relatively small number of hypotheses compared with most previous simulation results in the literature. Our results show that the online testing framework offers a smaller advantage in terms of power properties in this small *N* setting compared to when a very large number (≥ 1000) of hypotheses are tested. However, in some clinical trial settings (e.g. testing for interaction with genomic data) there can be a large number of hypotheses and a desire for FDR control (as opposed to FWER control), and so online error rate control may be especially applicable.

As noted in Section 4, the relative performance of the online algorithms compared with Bonferroni and Uncorrected testing crucially depends on the number of effective treatments, the order in which they are tested in the trial, as well as the assumed upper bound *N*_bound_. Hence, the ‘best’ online algorithm to use may be quite different depending on the trial context and goals. In general, across the simulation scenarios for the fully online setting, we see that LOND seems to offer quite a good compromise by guaranteeing at least as many rejections as Bonferroni and often seeing noticeable increases in sensitivity, while still maintaining (depending on the trial context) reasonable FWER levels. In the online batched setting however, BatchPRDS seems to be preferable to LOND as it has a similar or higher sensitivity and disjunctive power, while maintaining FWER control below the nominal level.

As seen in the case study results, in practice the online testing algorithms may not be ‘competitive’ in terms of the number of rejections when compared with Uncorrected testing unless a more relaxed *α* is used. However, the potential inflation on top of that relaxation for many of the online algorithms also needs to be taken into account. In that sense, BatchPRDS is particularly appealing due to the lack of further FWER inflation.

We have started with the simplifying assumption that each experimental treatment is tested exactly once, as soon as the outcomes from a pre-specified number of patients have been observed. A useful extension would be to allow multiple looks (stages) for each treatment arm, with early stopping for futility and efficacy. Indeed, the STAMPEDE trial included interim analyses that we did not take into account in our analysis in Section 5. A complicating factor is that to directly apply the online testing algorithms, we have only assigned the adjusted testing level *α_i_* to hypothesis *H_i_* at the point of testing. To allow repeated testing of hypothesis *H_i_* and early stopping, we would require *H_i_* to be determined in advance. For further discussion and proposals for this setting, we refer the interested reader to Zrnic et al. [23], who proposed a framework for asynchronous online testing. As well, recent work by Zehetmayer et al. [36] shows how to use LOND specifically for group-sequential platform trials.

Another assumption that we have made is that *N*_bound_ ≥ *K*, i.e. that we never test more treatment arms than originally planned for. In the case where *N*_bound_ < *K*, then a simple method of accommodating this change has been proposed by [17, pg. 10], but the power properties have not been explored in the context of platform trials. We could also make further comparisons of online testing with existing methodologies for controlling the FWER when adding treatment arms to a platform trial [37, 38]. Finally, one limitation of our investigation is that in practice, future comparisons depend on results of past trials, which in turn depend on which online testing algorithm is being used. We did not account for this in our simulations. Taking this into consideration would be highly complex, but may be a useful avenue for future research.

Alternative type I error rates to the FWER and FDR have been proposed in the literature. One example is the Error Rate per Family (ERpf) proposed by Tukey [39]. The ERpF is defined as the number of type I errors divided by the number of families of hypotheses. As pointed out by an anonymous reviewer, while the FWER may be appropriate for multiplicity within a study, the ERpF is meant for multiplicity control across studies. Hence, if one can view a platform trial as a series of distinct studies (e.g. corresponding to a change to the standard-of-care), the ERpF may be a more appropriate metric to control. The ERpF can be controlled exactly across studies by using what is called an additive multiplicity correction [40]. As future research, it would be interesting to compare online control of the FWER or FDR with ERpF control. It would also be interesting to explore whether recently proposed methods of FDR control using e-values [41] could be applied to the platform trial setting.

As mentioned in the introduction, the need for multiplicity adjustments for platform trials is controversial, and there are some platform trial contexts where applying such adjustments may be difficult and/or undesirable. For example, platform trials often involve multiple pharmaceutical sponsors and a natural question to ask is why a sponsor would participate in a platform trial that uses *any* multiplicity adjustment, given that they can conduct their own two-arm trial with *no* multiplicity adjustment. To help address this issue, one partial solution is to adjust for multiplicity (using online testing or otherwise) at a level *α* that is higher than the usual 2.5% (one-sided). As shown in the case study in Section 5, this can result in adjusted significance levels above 2.5%. More generally, the efficiencies that a platform trial bring (e.g. in terms of time and cost of recruiting control patients) may still mean that a sponsor would be willing to join a trial even if there is a price to be paid in terms of multiplicity adjustment. Meanwhile, in the context of emerging infectious diseases, there is a real need to quickly find a treatment that works and it is unclear how the use of multiplicity adjustment may affect that time. A fuller discussion around multiplicity adjustment (and online testing more specifically) for platform trials would be very useful, but is out of scope of this paper.

## Supplementary Material

Supporting Information

## Figures and Tables

**Table 1:** Overview of simulation scenarios and performance metrics.

Treatment means	Arm entry times	Algorithms	Performance metrics
Global null	All arms at once	Bonferroni, ADDIS-spending	FWER and FDR
Fixed means	Batches of size 5	ADDIS, LORD, SAFFRON, BH	Disjunctive power
Staircase scenario	Staggered starts	BatchBH, BatchPRDS, BatchStBH	Sensitivity
	One-by-one		

**Table 2:** Reported results for the STAMPEDE trial. SOC = Standard-of-care.

Trial arm	*p*-value
**B**: SOC + zoledronic acid	0.450
**C**: SOC + docetaxel	0.006
**E**: SOC + zoledronic acid + docetaxel	0.022
**D**: SOC + celecoxib	0.847
**F**: SOC + zoledronic acid + celecoxib	0.130
**G**: SOC + abiraterone	0.001
**H**: SOC + radiotherapy	0.266

**Table 3:** Rejections and current significance level *α*_8_ of different algorithms using the results of the STAMPEDE trial, with the ordering as in Table 2.

Algorithm	Hypotheses rejected			*α*_8_		
	*α* = 0.025	*α* = 0.05	*α* = 0.1	*α* = 0.025	*α* = 0.05	*α* = 0.1
Uncorrected	C, E, G	C, E, G	C, E, G	0.0250	0.0500	0.1000
Bonferroni	G	G	G	0.0013	0.0025	0.0050
ADDIS-spending	G	G	G	0.0005	0.0011	0.0021
BH	C, G	C, G	C, E, G	–	–	–
ADDIS	–	G	G	0.0003	0.0016	0.0031
SAFFRON	G	C, G	C, E, G	0.0041	0.0165	0.0412
LORD	–	–	–	0.0001	0.0002	0.0003
LOND	G	G	G	0.0025	0.0050	0.0100
BatchBH	G	C, G	C, E, G	0.0019	0.0057	0.0151
BatchPRDS	G	C, G	C, E, G	0.0019	0.0057	0.0151
BatchStBH	C, G	C, E, G	C, E, G	0.0381	0.1015	0.1238

## Data Availability

All of the data that support the findings of this study are available within the paper itself.
