# Supplementary material for: Online error rate control for platform trials
Source: Stat Med. 2023 Apr 2;42(14):2475–95. doi: 10.1002/sim.9733 (PMC7614610; doi:10.1002/sim.9733)
Supplement: Supplementary file 1 — Appendix S1: Supporting Information [file SIM-42-2475-s001.pdf]

Supporting information for "Online error rate control for platform trials"

David S. Robertson*[a], James M. S. Wason[b], Franz König[c], Martin Posch[c], and Thomas Jaki[a,d]

[a]MRC Biostatistics Unit, School of Clinical Medicine, University of Cambridge, UK

[b]Population Health Sciences Institute, Faculty of Medical Sciences, Newcastle University, UK

[c]Section of Medical Statistics, Medical University of Vienna, Austria

[d]Faculty of Informatics and Data Science, University of Regensburg, Regensburg, Germany

A Online testing algorithms implementation

For all the simulations, we use the `onlineFDR` R package in order to implement the online testing algorithms. Table A1 below gives the parameter values used for each of the algorithms. All of the γ_i sequences are chosen so that $\sum_{i=1}^{N_{\text{bound}}} \gamma_i = 1$.

**Algorithm**	γ_i **sequence**	**Other parameters**
ADDIS-spending	$\gamma_i \propto 1/i^{1.6}$	$\lambda = 0.25$, $\tau = 0.5$
ADDIS	$\gamma_i \propto 1/i^{1.6}$	$\lambda = \tau = 0.5$, $w_0 = \lambda\tau\alpha/2$
LOND	$\gamma_i \propto 1$	
LORD	$\gamma_i \propto \frac{\log(\max(i,2))}{i \exp(\sqrt{\log(i)})}$	$w_0 = \alpha/10$, $b_0 = \alpha - w_0$
SAFFRON	$\gamma_i \propto 1/i^{1.6}$	$\lambda = 0.5$, $w_0 = \alpha/2$
BatchBH	$\gamma_i \propto 1/i^{1.6}$	
BatchPRDS	$\gamma_i \propto 1/i^{1.6}$	
BatchStBH	$\gamma_i \propto 1/i^{1.6}$	$\lambda = 0.5$

Table A1: Parameter values used for the online testing algorithms.

*david.robertson@mrc-bsu.cam.ac.uk

B Additional simulation results

B.1 Global null

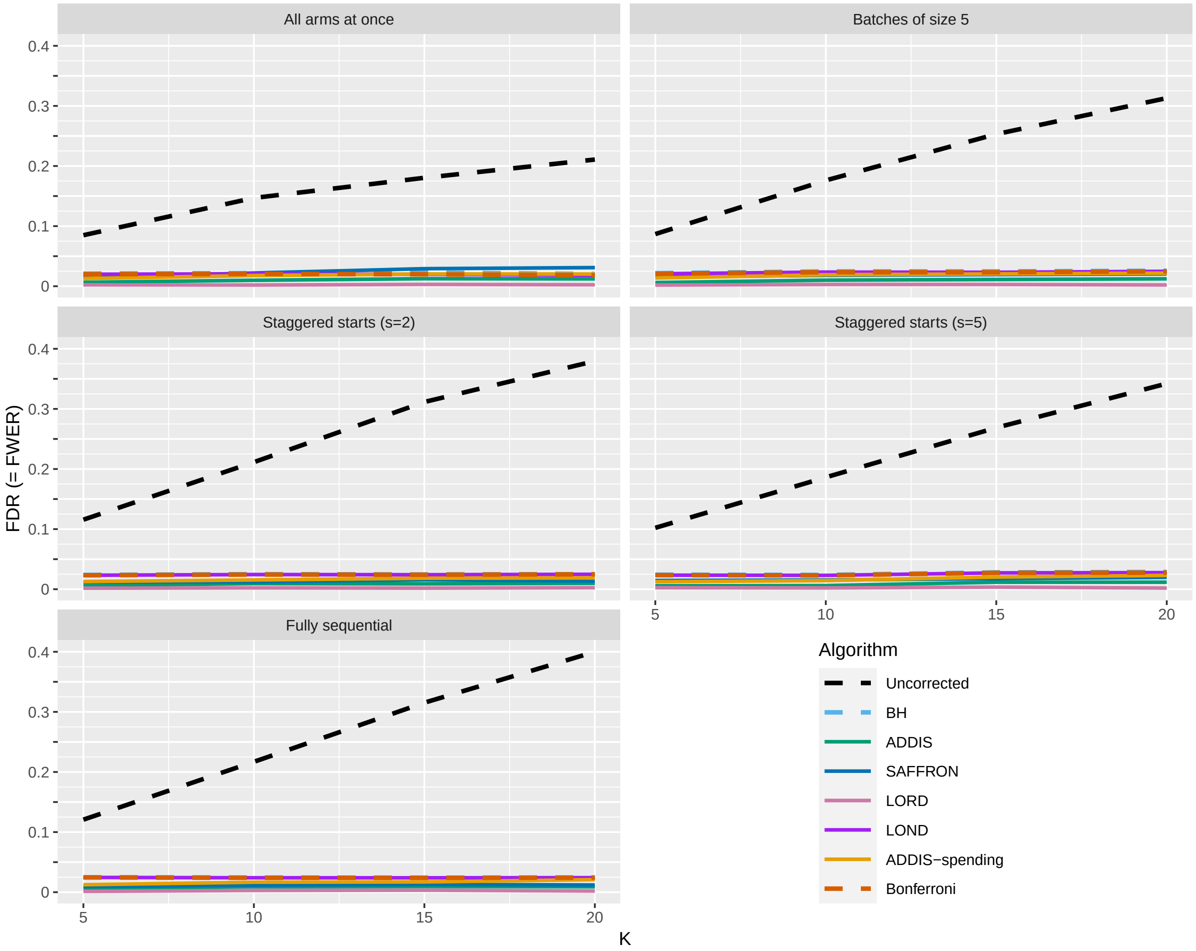

Figure B1: FDR (= FWER) under the global null with varying patterns of arm entry times and $N_{\text{bound}} = K$.

B.2 Fully sequential setting

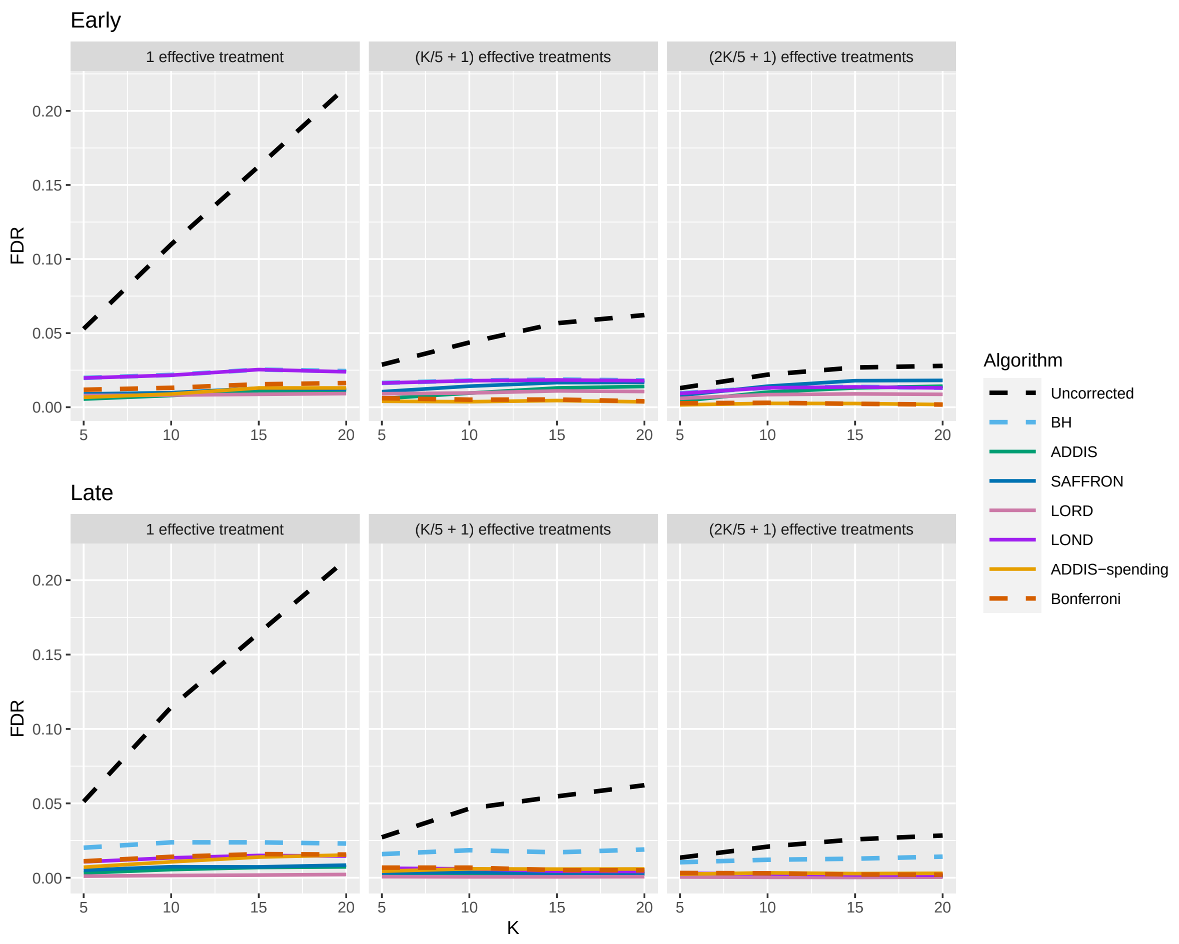

Figure B2: FDR for fixed means and different numbers of effective treatments, with $N_{\text{bound}} = K$.

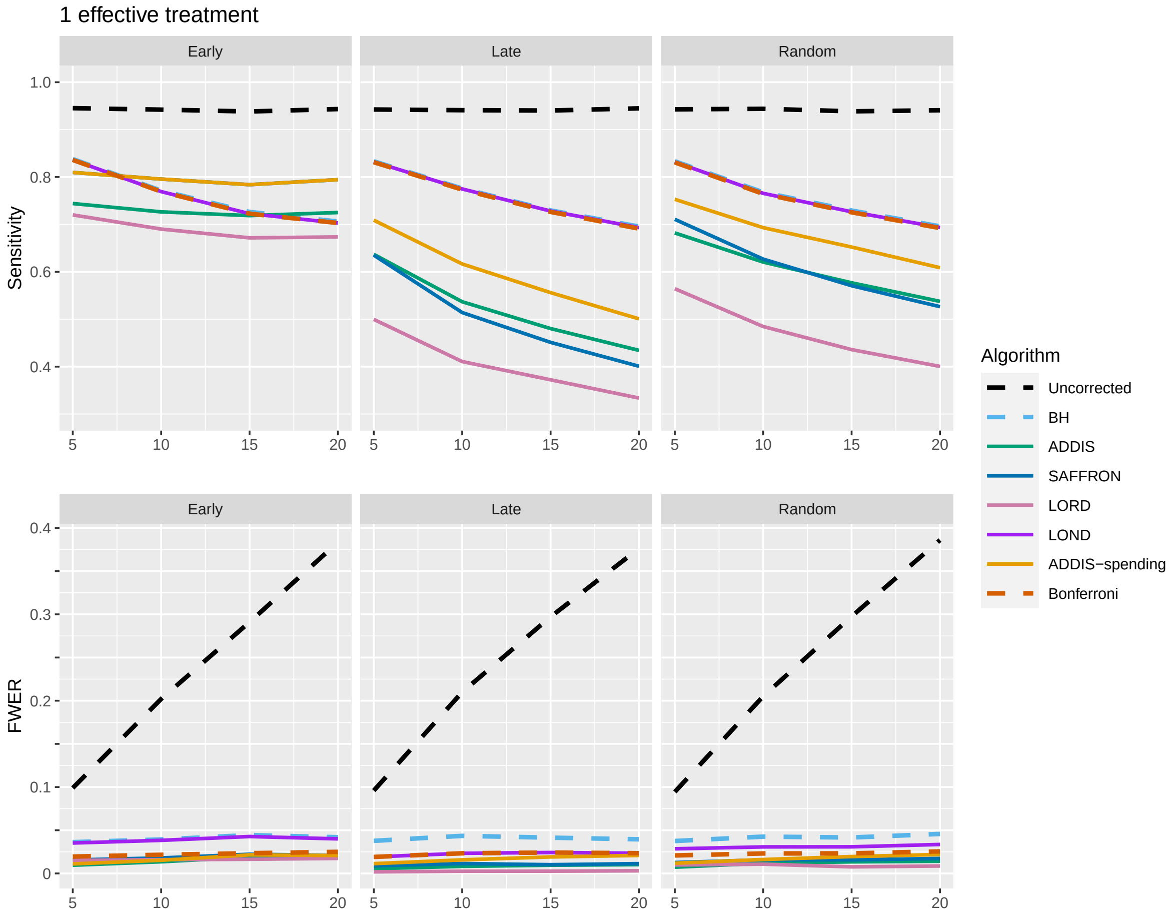

Figure B3: Sensitivity and FWER for fixed means and 1 effective treatments, with $N_{\text{bound}} = K$.

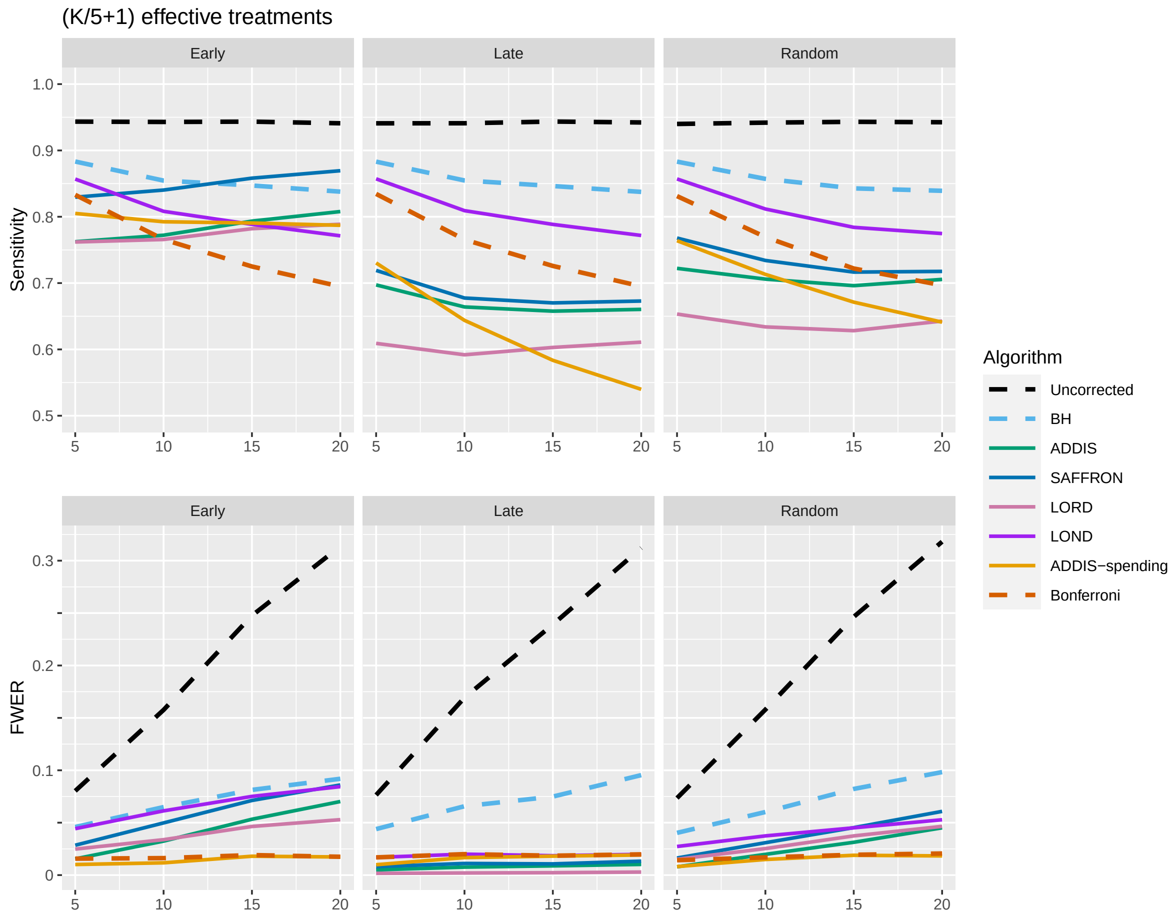

Figure B4: Sensitivity and FWER for fixed means and $(K/5 + 1)$ effective treatments, with $N_{\text{bound}} = K$.

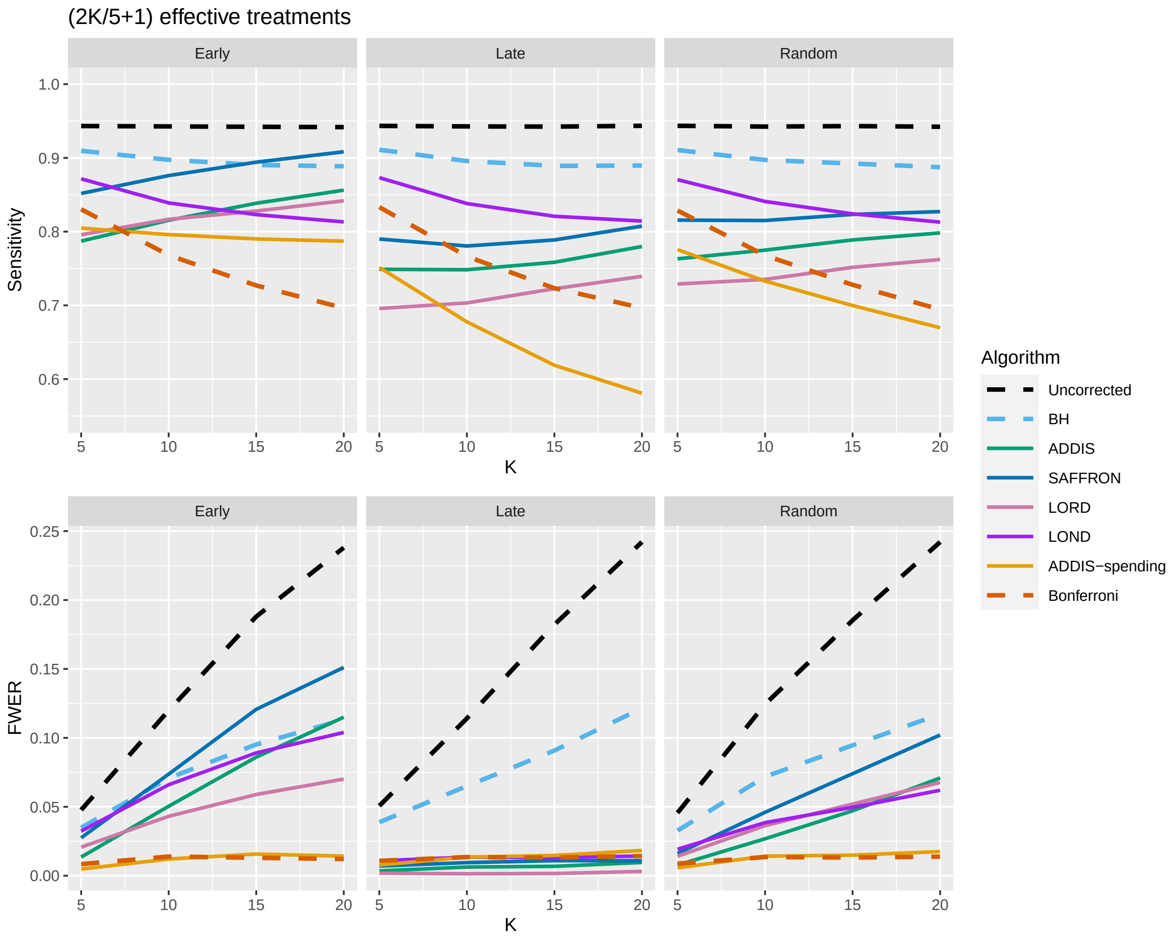

Figure B5: Sensitivity and FWER for fixed means and $(2K/5+1)$ effective treatments, with $N_{\text{bound}} = K$.

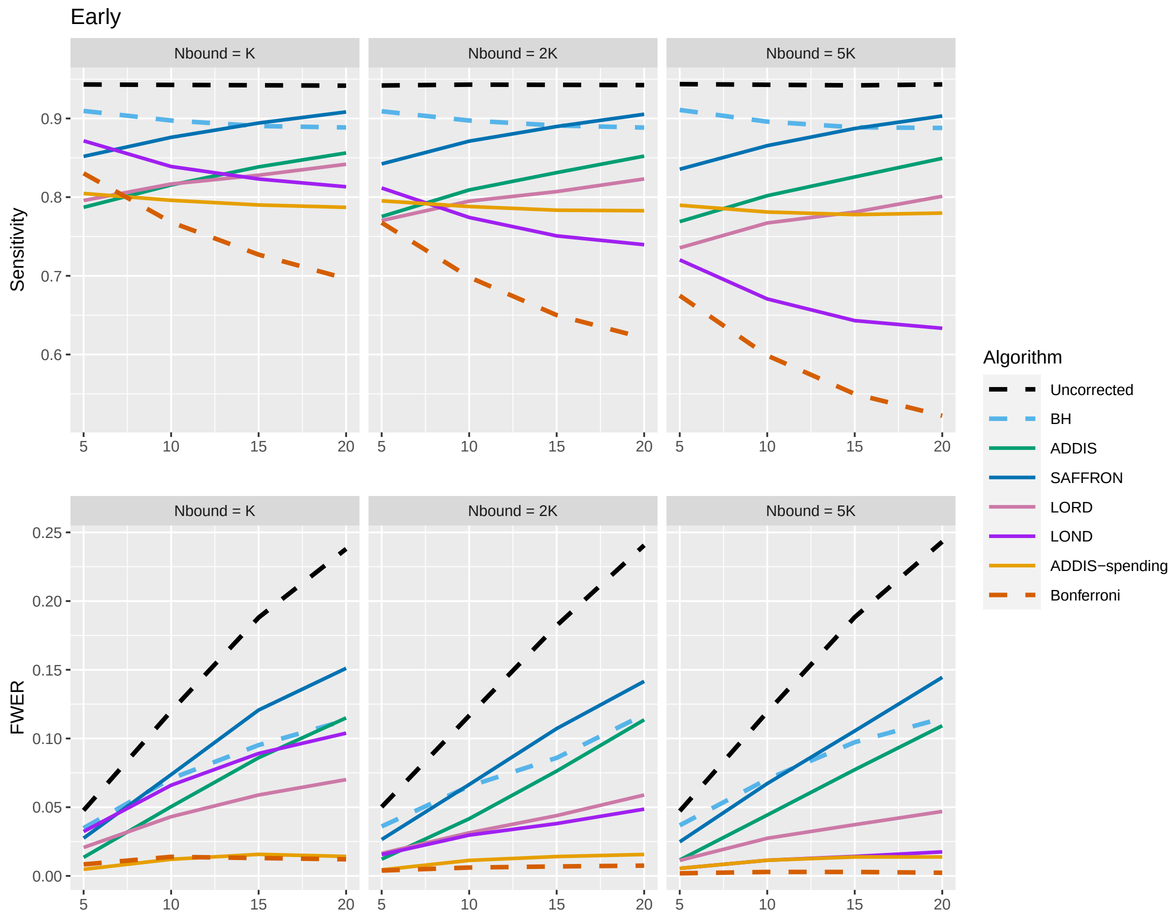

Figure B6: Best case sensitivity with corresponding FWER for fixed means and $(2K/5 + 1)$ effective treatments, with varying N_{bound}.

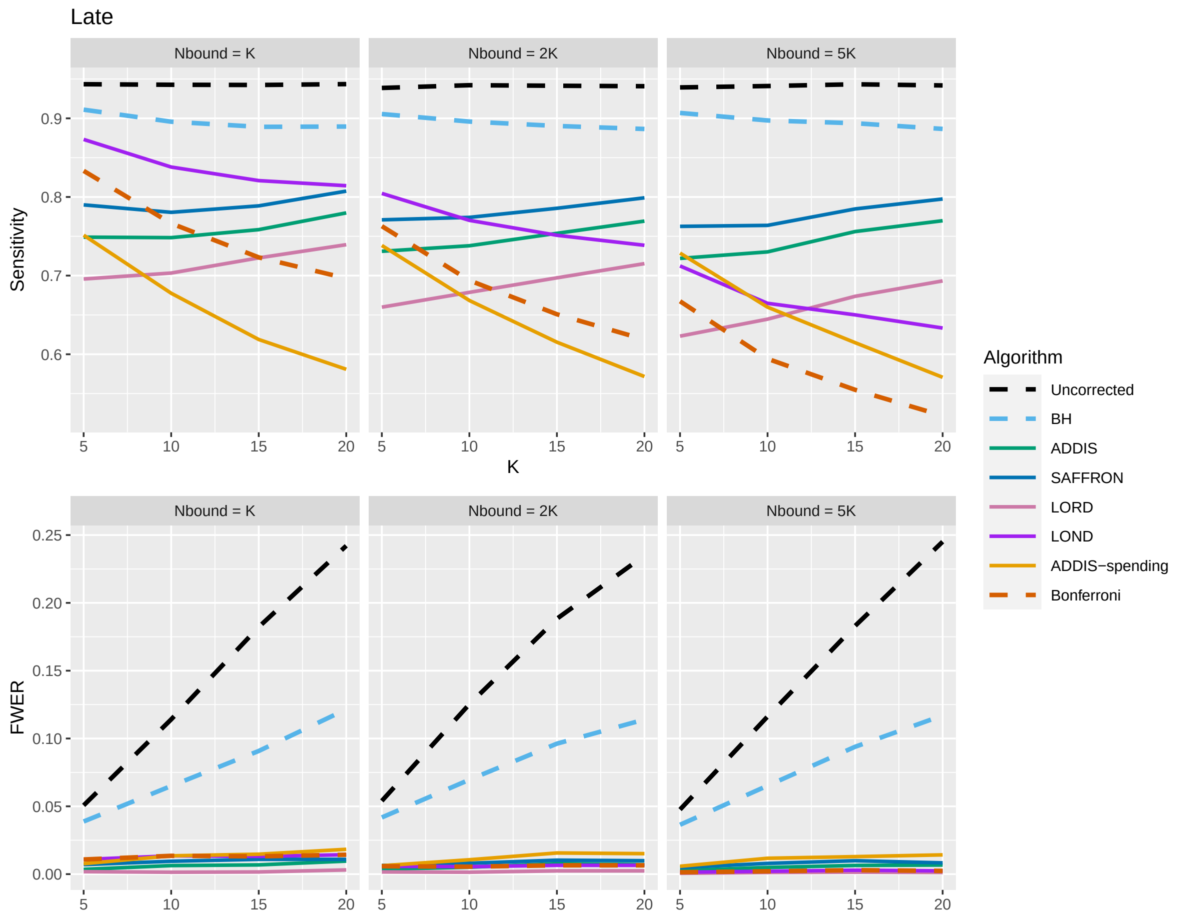

Figure B7: Worst case sensitivity with corresponding FWER for fixed means and $(2K/5 + 1)$ effective treatments, with varying N_{bound}.

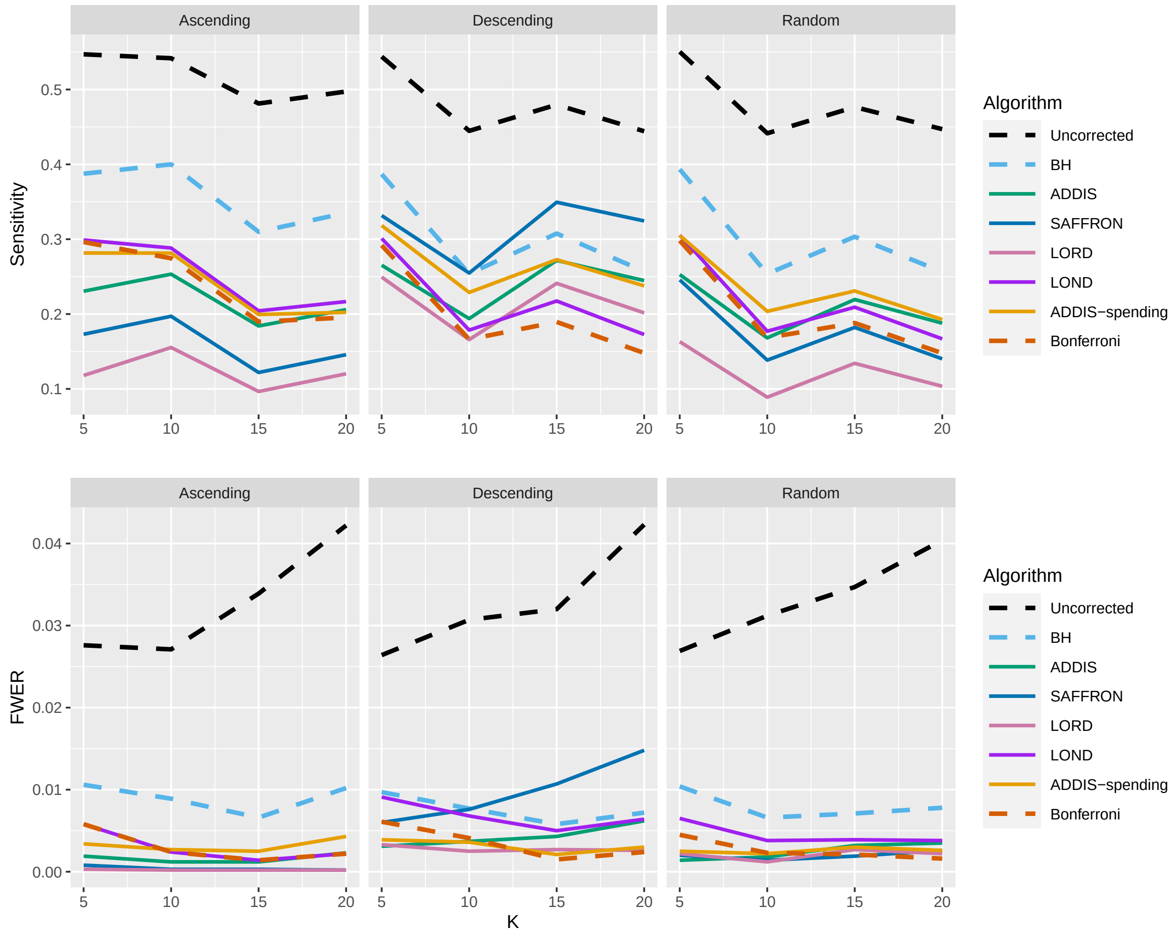

Figure B8: Sensitivity and FWER for the staircase scenarios. Here $N_{\text{bound}} = 2K$ for the sensitivity plots and $N_{\text{bound}} = K$ for the FWER plots.

B.3 Batched algorithms

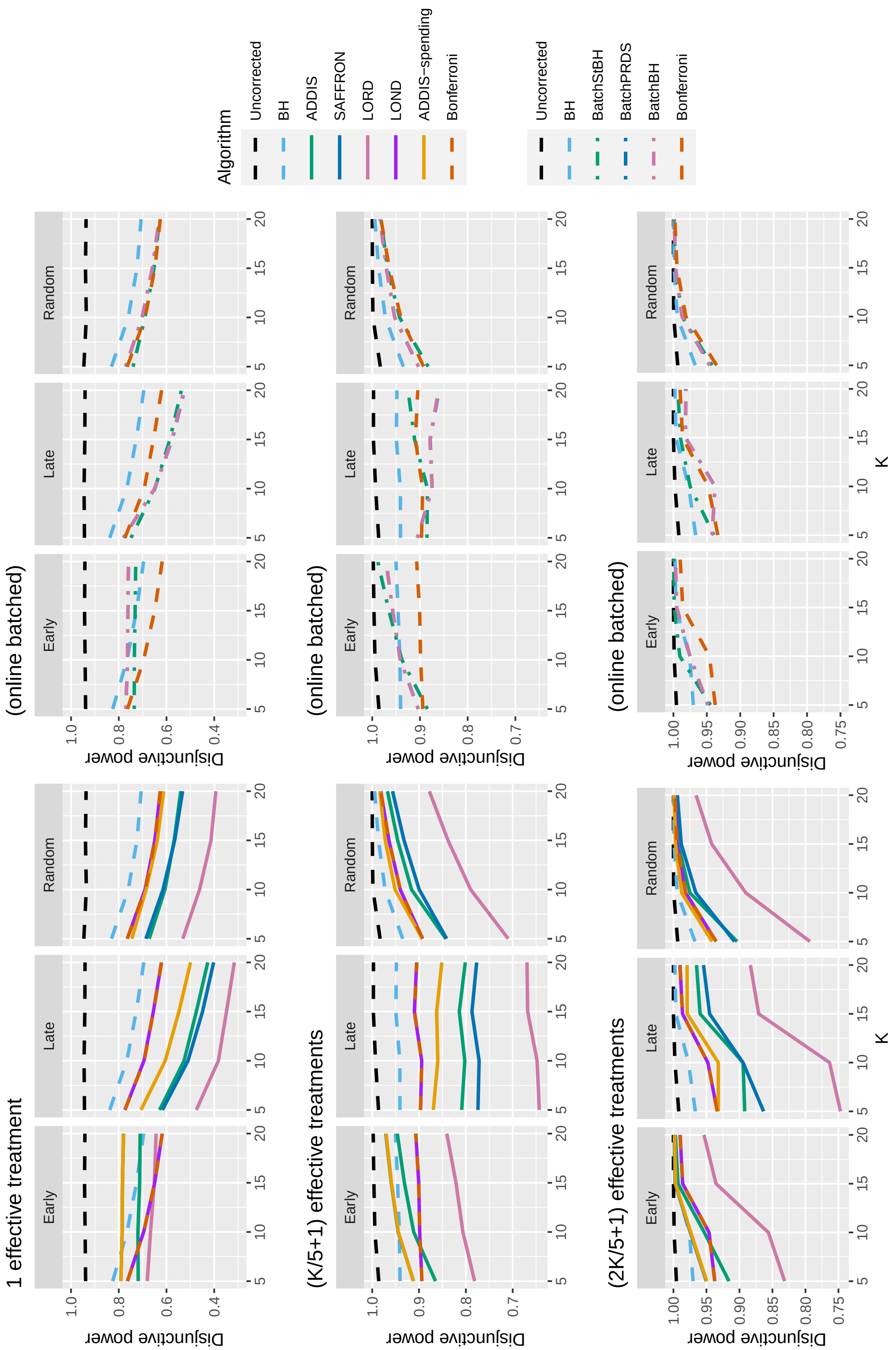

Figure B9: Comparison of the disjunctive power for fully sequential and batched online algorithms, with $N_{\text{bound}} = 2K$.

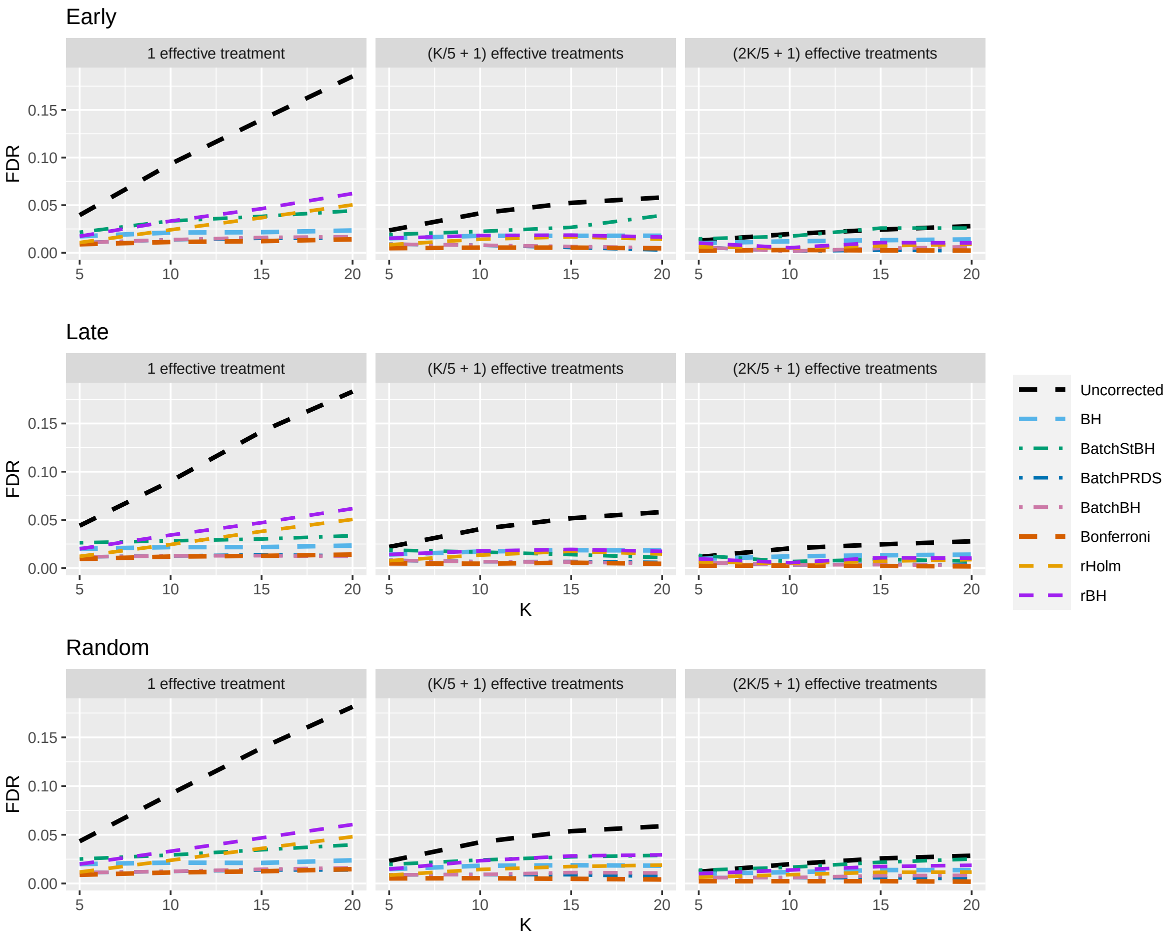

Figure B10: FDR for the online batched algorithms, with $N_{\text{bound}} = K$. rBH = repeated application of BH, rHolm = repeated application of Holm.

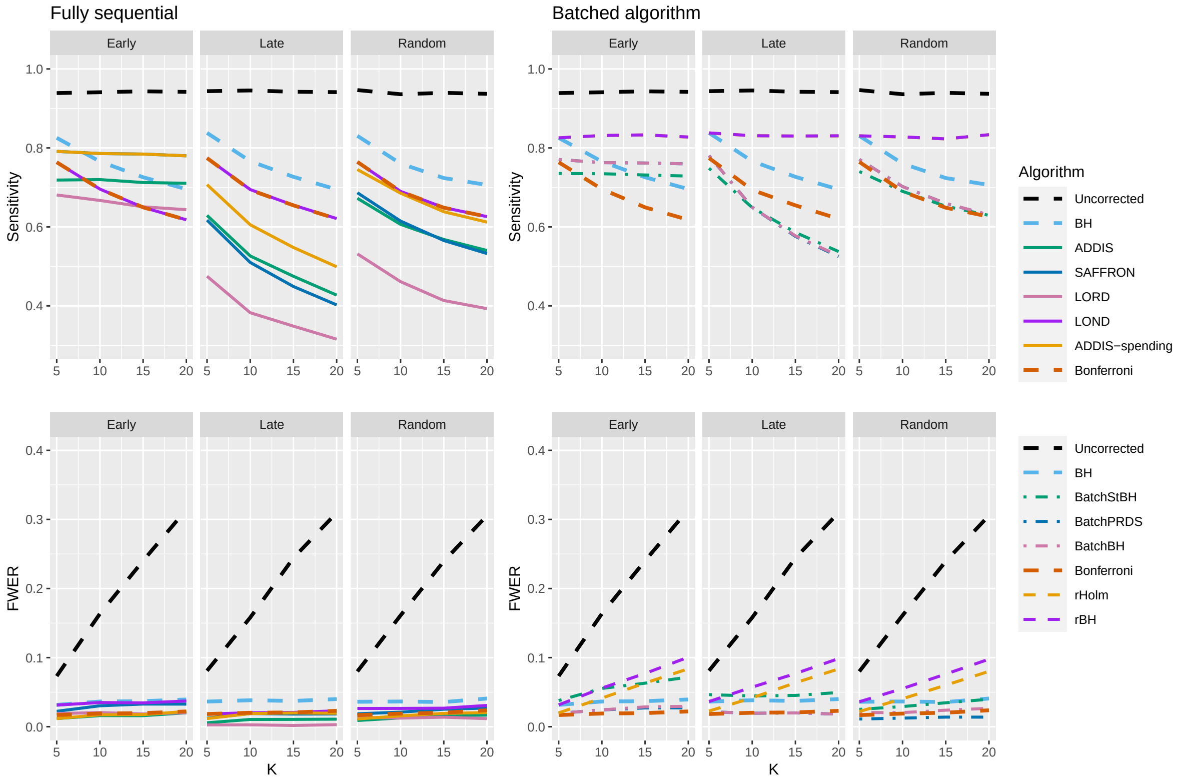

Figure B11: Comparison of sensitivity and FWER for fully online and online batched algorithms, with 1 effective treatment. Here $N_{\text{bound}} = 2K$ for the sensitivity and $N_{\text{bound}} = K$ for the FWER. rBH = repeated application of BH, rHolm = repeated application of Holm.

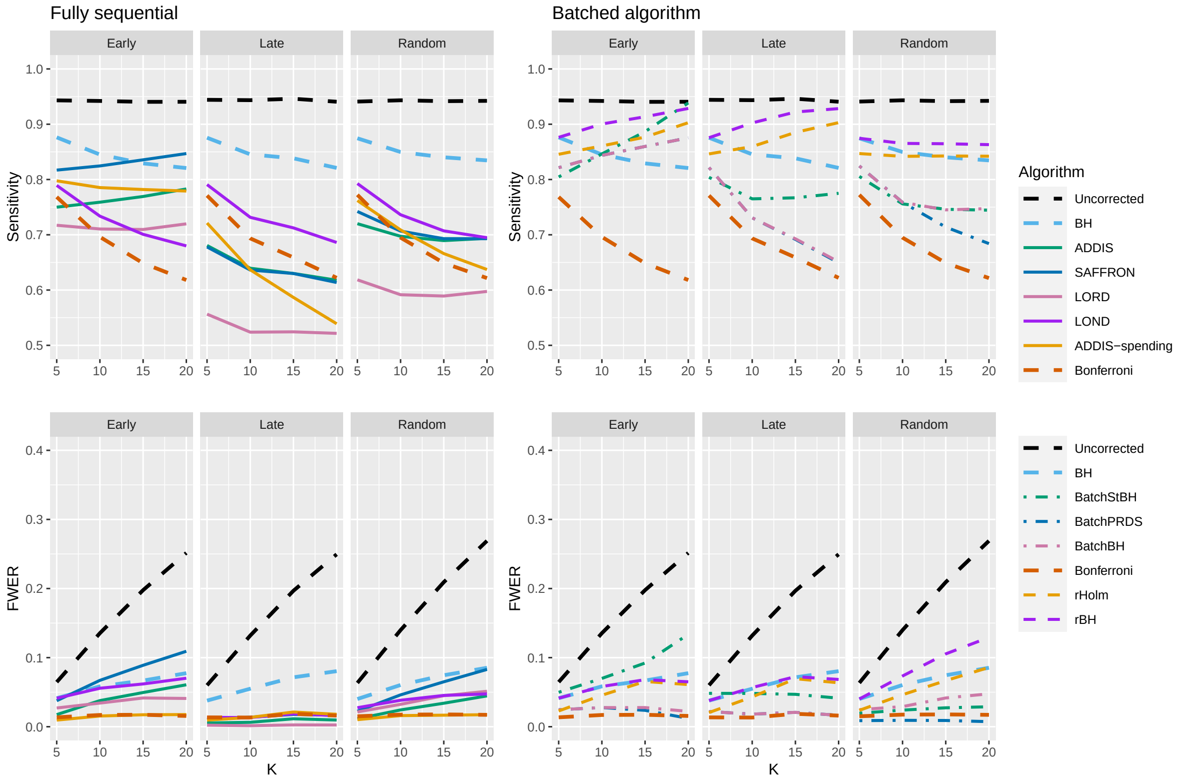

Figure B12: Comparison of sensitivity for fully online and online batched algorithms, with $(K/5+1)$ effective treatments. Here $N_{\text{bound}} = 2K$ for the sensitivity and $N_{\text{bound}} = K$ for the FWER. rBH = repeated application of BH, rHolm = repeated application of Holm.

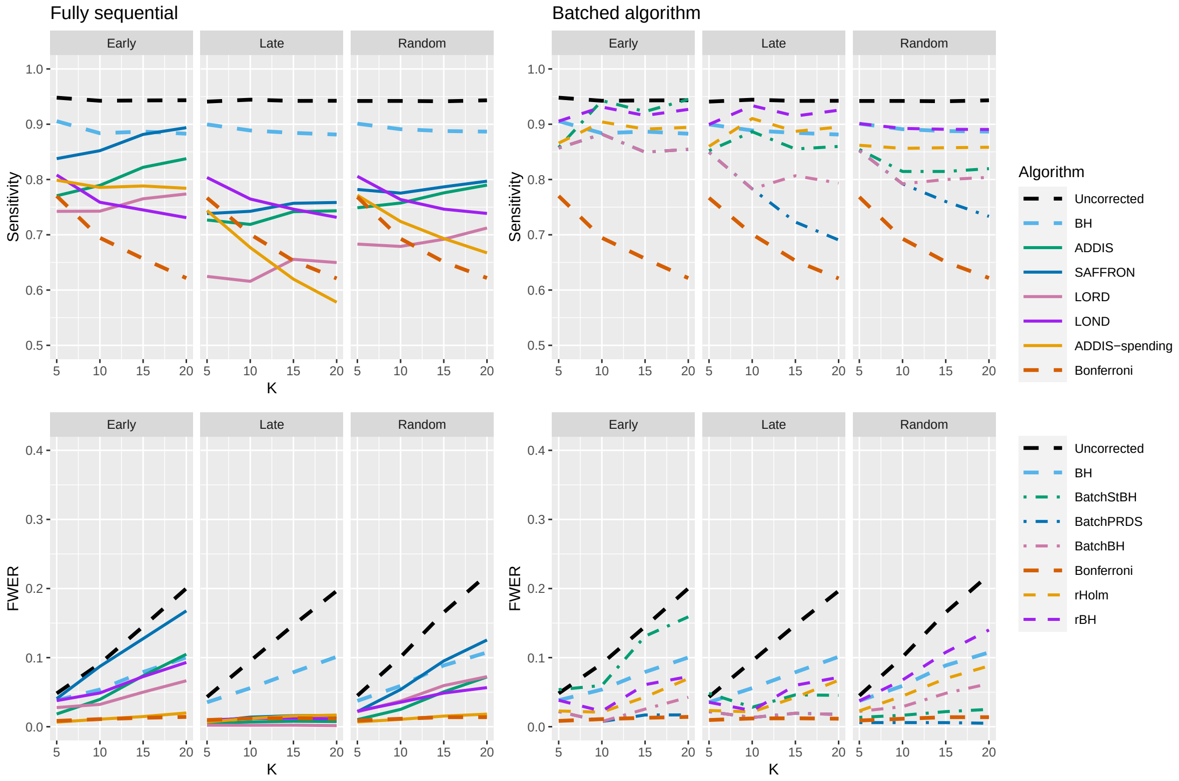

Figure B13: Comparison of sensitivity for fully online and online batched algorithms, with $(2K/5+1)$ effective treatments. Here $N_{\text{bound}} = 2K$ for the sensitivity and $N_{\text{bound}} = K$ for the FWER. rBH = repeated application of BH, rHolm = repeated application of Holm.

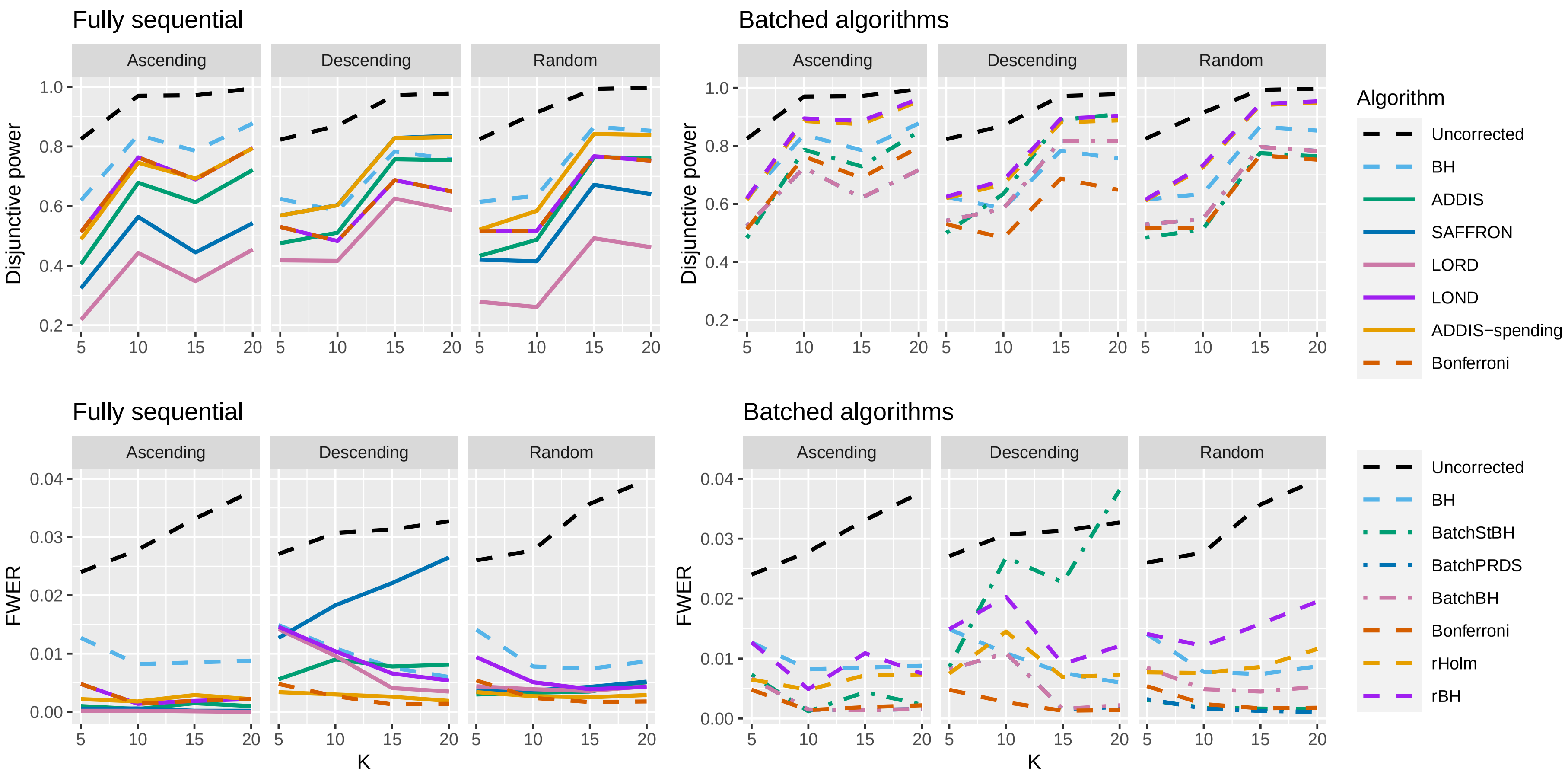

Figure B14: Comparison of the FWER ($N_{\text{bound}} = K$) and disjunctive power ($N_{\text{bound}} = 2K$) for fully online and online batched algorithms under the staircase scenario. rBH = repeated application of BH, rHolm = repeated application of Holm.

C Case study: STAMPEDE trial

Table C2 shows the results if the ordering of treatment arms B and C within the first batch were switched. The results are similar as before, except that ADDIS-spending and ADDIS now reject hypotheses C and G when $\alpha \in \{0.05, 0.1\}$ and $\alpha = 0.1$, respectively.

**Algorithm**	**Hypotheses rejected**			$\boldsymbol{\alpha_8}$		
	$\alpha = 0.025$	$\alpha = 0.05$	$\alpha = 0.1$	$\alpha = 0.025$	$\alpha = 0.05$	$\alpha = 0.1$
Uncorrected	C, E, G	C, E, G	C, E, G	0.0250	0.0500	0.1000
Bonferroni	G	G	G	0.0013	0.0025	0.0050
ADDIS-spending	G	**C**, G	**C**, G	0.0005	0.0011	0.0021
BH	C, G	C, G	C, E, G	–	–	–
ADDIS	–	G	**C**, G	0.0003	0.0016	0.0062
SAFFRON	G	C, G	C, E, G	0.0041	0.0165	0.0412
LORD	–	–	–	0.0001	0.0002	0.0003
LOND	G	G	G	0.0025	0.0050	0.0100
BatchBH	G	C, G	C, E, G	0.0019	0.0057	0.0151
BatchPRDS	G	C, G	C, E, G	0.0019	0.0057	0.0151
BatchStBH	C, G	C, E, G	C, E, G	0.0381	0.1015	0.1238

Table C2: Rejections and current significance level α_8 of different algorithms using the results of the STAMPEDE trial, with the ordering of treatment arms B and C switched. The new rejections are shown in bold font.
